# Identifying performance factors of long-term care facilities in the context of the COVID-19 pandemic: a scoping review protocol

**DOI:** 10.1186/s13643-022-02069-1

**Published:** 2022-09-23

**Authors:** Josiane Létourneau, Emilie Bélanger, Drissa Sia, Idrissa Beogo, Stephanie Robins, Katya Kruglova, Maripier Jubinville, Eric Nguemeleu Tchouaket

**Affiliations:** 1 Department of Nursing, Université du Québec en Outaouais, St-Jérôme Campus, 5, rue Saint-Joseph, Office J-2204, Saint-Jérôme, Québec, Canada; 2 School of Nursing, Faculty of Health Sciences, University of Ottawa, Ottawa, Ontario Canada

**Keywords:** Long-term care facilities, Performance factors, COVID-19, Scoping review, Residents, Facilitators, Barriers, Conceptual framework

## Abstract

**Background:**

Long-term care facilities (LTCFs) have been severely affected by the COVID-19 pandemic with serious consequences for the residents. Some LTCFs performed better than others, experiencing lower case and death rates due to COVID-19. A comprehensive understanding of the factors that have affected the transmission of COVID-19 in LTCFs is lacking, as no published studies have applied a multidimensional conceptual framework to evaluate the performance of LTCFs during the pandemic. Much research has focused on infection prevention and control strategies or specific disease outcomes (e.g., death rates). To address these gaps, our scoping review will identify and analyze the performance factors that have influenced the management of COVID-19 in LTCFs by adopting a multidimensional conceptual framework.

**Methods:**

We will query the CINAHL, MEDLINE (Ovid), CAIRN, Science Direct, and Web of Science databases for peer-reviewed articles written in English or French and published between January 1, 2020 and December 31, 2021. We will include articles that focus on the specified context (COVID-19), population (LTCFs), interest (facilitators and barriers to performance of LTCFs), and outcomes (dimensions of performance according to a modified version of the *Ministère de la santé et des services sociaux du Québec* conceptual framework). Each article will be screened by at least two co-authors independently followed by data extraction of the included articles by one co-author and a review by the principal investigator.

**Results:**

We will present the results both narratively and with visual aids (e.g., flowcharts, tables, conceptual maps).

**Discussion:**

Our scoping review will provide a comprehensive understanding of the factors that have affected the performance of LTCFs during the COVID-19 pandemic. This knowledge can help inform the development of more effective infection prevention and control measures for future pandemics and outbreaks. The results of our review may lead to improvements in the care and safety of LTCF residents and staff.

**Scoping review registration:**

Research Registry researchregistry7026

**Supplementary Information:**

The online version contains supplementary material available at 10.1186/s13643-022-02069-1.

## Background

In March 2020, the World Health Organization declared COVID-19 a pandemic and emphasized their concern over the severity of the virus [1]. Since then, the disease has disproportionately affected long-term care facilities (LTCFs) around the world, causing excess morbidity and mortality in residents [2, 3]. As of October 2020, over half of the COVID-19 deaths throughout Europe occurred among LTCF residents [4]. By the end of August 2020, 42% of all COVID-19 deaths in the USA were among LTCF residents, who represented only 0.6% of the population [5]. Deaths due to COVID-19 among Canadian LTCF residents made up nearly 80% of all COVID-19 deaths between March 2020 and July 2020 [6]. In the province of Québec, 92% of the COVID-19 deaths were among individuals aged 70 and older, of whom 64% were living in LTCFs [7].

To date, within research related to COVID-19, the emphasis has been on publishing guidelines [8], discussing mitigation strategies [9, 10], and reporting specific outcomes such as COVID-19 death rates and case numbers within LTCFs. Only one study [11] discusses performance in LTCFs within the context of COVID-19, defining performance based on a ranking system created by the Centers for Medicare & Medicaid Services that considered three domains: health inspections, quality measures, and nurse staffing. A few studies have attempted to identify factors that may have contributed to the poor COVID-19 outcomes in LTCFs, describing both barriers and facilitators related to the management of the pandemic. On the one hand, an inadequate staff-to-resident ratio has resulted in overtime hours for staff and, as a consequence, worse care for residents [12, 13]. On the other hand, the government’s timely response and the implementation of mandatory infection prevention and control (IPC) measures both in LTCFs and their surrounding communities helped curb the transmission of the virus [6, 13]. Furthermore, LTCFs with a lower crowding index (i.e., average number of residents per room and bathroom) and those with greater funding and more hours of direct care had fewer COVID-19 deaths among residents [14, 15]. In addition, qualitative research has provided insight into the performance factors that limited the spread of COVID-19 in LTCFs, including early identification and management of cases, the testing of asymptomatic residents and staff, and organizational factors such as leadership and IPC staff training [12]. Facilities that implemented these strategies were more successful at mitigating the spread of the virus. Though several factors affecting COVID-19 outcomes in LTCFs have been described, a comprehensive review of these factors has yet to be undertaken to understand their impact on the relative performance of LTCFs during the pandemic [4, 15]. This and the significant human impact on both residents and staff of LTCFs caused by the COVID-19 pandemic are the motivation behind this scoping review.

Our preliminary search of the CINAHL and MEDLINE databases identified several articles examining the performance of LTCFs during the COVID-19 pandemic. Thompson et al. [16] surveyed COVID-19 death rates globally and discussed potential causes (e.g., inadequate staff training and personal protective equipment) and lack of prevention strategies (e.g., resident screening), but recommendations were limited in scope and detail. Chen et al. [17] reviewed the US government’s policies aimed at mitigating COVID-19 transmission in LTCFs in order to facilitate the development of future evidence-based regulations. Though this review detailed the implemented policy changes, it did not investigate their impact on the management of the pandemic in LTCFs. A scoping review by Giri et al. [18] explored the facilitators of the spread of COVID-19 within LTCFs; yet, this review searched only one database and did not define performance. To our knowledge, no scoping review has examined the performance factors that have influenced the management of COVID-19 in LTCFs using a conceptual framework.

## Aims and objectives

Accordingly, the aim of our scoping review is to identify and analyze the performance factors that have influenced the management of the COVID-19 pandemic in LTCFs using a multidimensional conceptual framework. Specifically, we aim to address the following objectives:

1. Identify the facilitators reported to have influenced the performance of LTCFs since the beginning of the COVID-19 pandemic
2. Identify the barriers faced by LTCFs reported to have influenced the performance of LTCFs since the beginning of the COVID-19 pandemic
3. Identify the gaps in the existing literature and the most pressing questions for future research.

## Methods

### Methodological framework

In conducting this scoping review, we will adhere to the framework developed by the Joanna Briggs Institute (JBI), which expands on previous work by Arksey and O’Malley as well as Levac et al. [19, 20]. The JBI framework includes nine steps: (1) formulating and aligning the review’s objective(s) and question(s); (2) developing the inclusion criteria in alignment with the objective(s) and question(s); (3) describing the approach to database queries, article selection, data extraction, and presentation of the findings; (4) searching for the evidence; (5) selecting the evidence; (6) extracting the evidence; (7) analyzing the evidence; (8) presenting the evidence; and (9) summarizing the evidence in relation to the objectives, drawing conclusions, and discussing any potential implications.

In the preparation of this protocol, we used the Preferred Reporting Items for Systematic Review and Meta-Analysis Protocols (PRISMA-P) checklist to ensure we included all necessary elements (see Additional file 1) [21]. We also consulted the Preferred Reporting Items for Systematic Reviews and Meta-Analyses Extension for Scoping Reviews (PRISMA-ScR), as it is tailored to scoping reviews (see Additional file 2) [22]. In addition, we followed the updated guidance for reporting scoping reviews outlined in the PRISMA 2020 Statement [23, 24].

### Conceptual framework

Though often expressed in terms of case and death rates [25, 26], the performance of LTCFs can be conceptualized in multiple ways. For example, the Organization for Economic Co-operation and Development framework defines performance as the successful attainment of goals at the lowest possible cost to the health care system [27], whereas the framework adopted by the Canadian Institute for Health Information assesses performance by responding to the questions “How healthy are Canadians?” and “How healthy is the health system?” [28]. In Québec, the *Cadre de référence ministériel d’évaluation de la performance du système public de santé et de services sociaux à des fins de gestion* framework developed by the *Ministère de la Santé et des Services Sociaux* (MSSS) conceptualizes performance within a healthcare system as the system’s capacity to reach its objectives with respect to the population’s health and well-being, while taking into account the optimization of the available resources and the accessibility and quality of the services provided [29]. Our team chose the MSSS framework for its multidimensional approach to performance evaluation.

We adopted the MSSS framework as the foundation for our scoping review’s conceptual framework of performance to guide data collection, analysis, synthesis, and presentation of evidence [30]. The MSSS framework includes three main dimensions of performance: (1) the accessibility of services, (2) the quality of services, and (3) the optimization of resources. The accessibility of services encompasses the sub-concepts of *equity* and *accessibility*; the quality of services *security*, *continuity*, and *efficacy*; and the optimization of resources *viability* and *efficiency*.

We modified the MSSS framework by adapting some of the sub-concepts or adding new ones to better fit our objectives and optimize database searches. Specifically, we found that the search results for *reactivity* retrieved by the CINAHL database did not correspond to the MSSS definition. Therefore, we replaced *reactivity* with the terms *adaptability* and *satisfaction*, whose definitions better corresponded to the MSSS definition of *reactivity*. For the same reason, we replaced the MSSS sub-concept of *viability* with the terms *resource management* and *resource mobilization*. In addition, the CINAHL terms *effectiveness* and *safety* were identified as synonyms of the MSSS sub-concepts *efficacy* and *security*, respectively, and thus were added to the final framework to ensure our database searches captured all relevant articles.

**Fig. 1:**
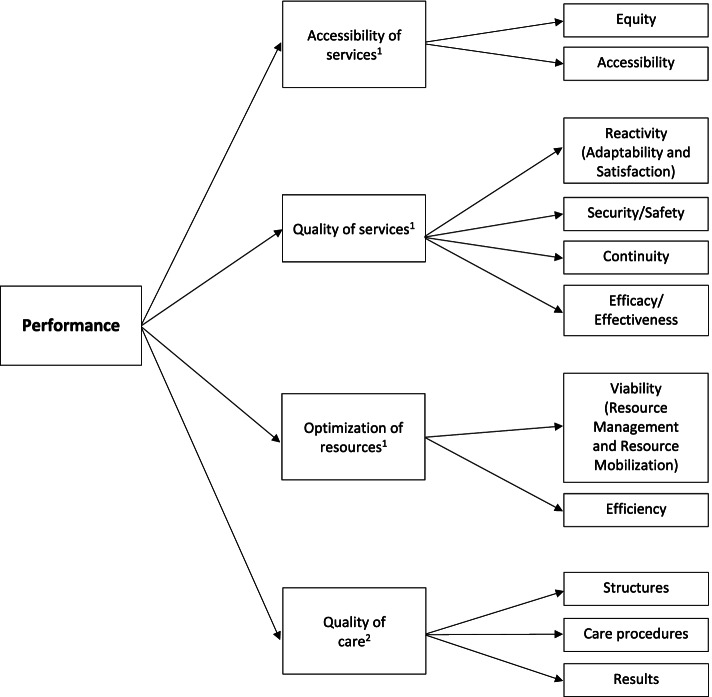
The adapted framework for the conceptualization of performance within a health care system. ^1^*Cadre de référence ministériel d’évaluation de la performance du système public de santé et de services sociaux à des fins de gestion* framework [29]. ^2^Elements from the *La prévention et le contrôle des infections nosocomiales* guidelines [31]

To ensure our performance framework incorporates IPC measures—which are not represented in the original MSSS framework—we added elements from the *La prévention et le contrôle des infections nosocomiales* guidelines [31]. These guidelines integrate organizational and physical factors of the healthcare environment, focusing on how the physical layout, resources, and structural elements (including staff members) of a healthcare setting can affect the quality of care related to IPC [31]. The adapted conceptual framework that will guide our review is displayed in Fig. 1.

Supplementary Information

### Inclusion criteria

The following are the inclusion criteria:

1. Population: LTCFs
2. Interest: factors (facilitators and barriers) influencing the performance of LTCFs
3. Comparison: not applicable
4. Outcome: performance of LTCFs
  - Accessibility of services
    i. Equity: ability to provide care and services according to need, without regard for personal characteristics (e.g., income, education, residential area)
    ii. Accessibility: ability to provide required care and services when and where they are needed
  - Quality of services
    i. Reactivity: ability to adapt to the expectations, values, and rights of residents (adaptability and satisfaction)
    ii. Security/safety: ability to minimize the risks for residents associated with interventions and the environment
    iii. Continuity: ability to provide the care and services required in an integrated and coordinated way
    iv. Effectiveness/efficacy: ability to improve the health and well-being of residents
  - Optimization of resources
    i. Viability: ability to respond to the current and future needs of the population, considering human, material, financial, technological, and informational resources (resource management and resource mobilization)
    ii. Efficiency: ability to use the available resources (human, material, financial, technological, and informational) optimally
  - Quality of care
    i. Structures: resources, physical layouts, and structural elements (committees, teams) that can directly or indirectly influence the quality of care in terms of IPC
    ii. Care procedures: standards and practices that underpin professional activities and the use of evidence-based IPC guidelines
    iii. Results: changes in the resident’s health status that may be attributed to the care and services received (e.g., nosocomial infection rates)
5. Time: COVID-19 pandemic timeframe (January 1, 2020 to December 31, 2021)

### Data sources and search strategy

This scoping review protocol was registered with the Research Registry (researchregistry7026: https://www.researchregistry.com/browse-the-registry#home/registrationdetails/6109a98e7fdaf6001ecffe67/).

In the Fall of 2021, our team will perform searches of the scientific literature using the five electronic databases: CINAHL, MEDLINE (Ovid), CAIRN, Science Direct, and Web of Science. Articles will be eligible if they meet our inclusion criteria in relation to the context (COVID-19), population (LTCFs), interest (facilitators and barriers to performance of LTCFs), and outcomes (dimensions of performance as per the adapted conceptual framework). We will restrict our searches to peer-reviewed articles written in English or French and published between January 1, 2020 and December 31, 2021. We will exclude articles that focused on infections other than COVID-19 and on health care settings other than LTCFs, as well as articles that reported on pharmaceutical treatments provided to LTCF residents and COVID-19 vaccination rates within LTCFs.

Two co-authors (JL, EB) will conduct independent database searches using the search strategies developed in collaboration with all team members. These search strategies are included as supplementary files (see Additional files 3, 4, 5, 6 and 7). The searches will be conducted using descriptors with the Boolean operators “AND” and “OR.” The results will be compared for consistency. The retrieved articles will be imported into the EndNote software, and all duplicates will be removed.

### Selection process

**Fig. 2:**
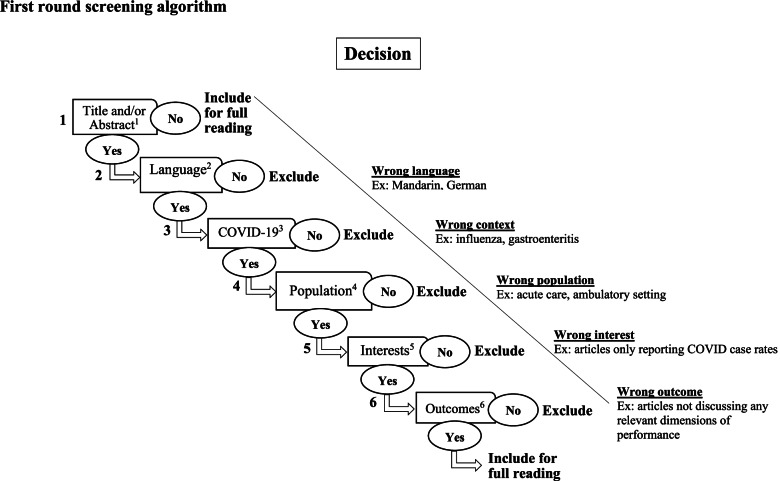
First round screening algorithm, adapted from previous work by Tchouaket et al. ^1^The reference has a title and/or an abstract. ^2^Language: the article is written in English or French. ^3^Context: COVID-19, coronavirus, 2019-nCoV, SARS-CoV-2, CoV-19, COVID. Excluded: all other infections (e.g., influenza, gastroenteritis). ^4^Population: long-term care facilities, nursing homes, assisted living facilities, homes for the aged, retirement homes, nursing homes, long-term care, and EHPAD. Excluded: acute care, hospital setting, and ambulatory setting. ^5^Interests: factors (facilitators and barriers) identified as affecting the performance of LTCFs. ^6^Outcomes: dimensions of performance (efficiency, effectiveness/efficacy, security/safety, accessibility, equity, continuity, adaptability, satisfaction, resource management, resource mobilization, structures, care procedures, results). Excluded: pharmaceutical research and vaccination studies

**Fig. 3:**
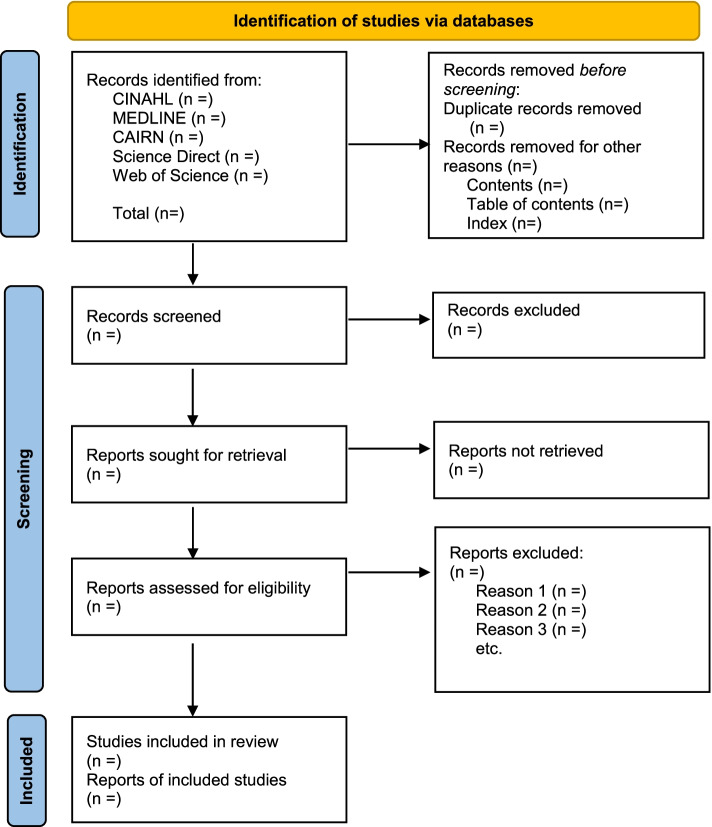
PRISMA flow chart, outlining the identification and selection stages of this review, adapted from *The PRISMA 2020 statement: an updated guideline for reporting systematic reviews* [23]. The term “report” signifies “a document (paper or electronic) supplying information about a particular study,” such as a journal article or government report, while the term “record” signifies “the title or abstract (or both) of a report indexed in a database or website” [23]

All articles will be exported to the Rayyan web platform [32] for the selection process, which will be completed using a screening algorithm (see Fig. 2) developed by the members of our team [3]. To ensure the reliability of the algorithm, a pilot test will be conducted where all co-authors will screen the titles and abstracts of the first 10% of the articles. The co-authors will then meet to discuss any discrepancies and decide if modifications to the algorithm or the overall screening process are necessary. Once the algorithm is tested and refined, the selection process will begin. First, half of the articles will be screened by two co-authors (JL, EB) with the other half divided among the remaining co-authors such that each article is screened by at least two co-authors. Article titles and abstracts will be screened for relevancy. If two co-authors deem an article relevant, it will be included. If one of the co-authors judges the article to be irrelevant, a third co-author will arbitrate. An article will be excluded if it is considered irrelevant by two co-authors. Second, all co-authors will read three of the selected articles in their entirety to verify their concordance with the inclusion criteria. The co-authors will then meet to address any issues that arose. All articles will then be read in their entirety by at least two co-authors (JL, EB), and if they satisfy the inclusion criteria, they will be included in the final scoping review. The selection process is displayed with a PRISMA flow chart (see Fig. 3).

### Data extraction

Data from the included articles will be extracted using a data charting form that will be developed by our team. The form will combine general items from a JBI template (e.g., year of publication) and more specific elements from the conceptual framework (e.g., dimensions of performance). The following information will be extracted from each article: title and abstract, author, year of publication, publication type, full citation, country of origin, study purpose, type of LTCF, population size (if applicable), study design, performance factors, and facilitators and barriers to performance. Prior to data extraction, the data charting form will be pilot tested following a process similar to that used for testing the screening algorithm: all co-authors will extract data from three articles and meet to resolve any issues and decide if any changes to the form are needed. Once the form is finalized, data from all included articles will be extracted by one co-author (EB) and reviewed by the principal investigator (JL).

### Analysis and presentation of the evidence

The results will be presented both narratively and with visual aids. An updated PRISMA flowchart will be provided, and descriptive characteristics (e.g., country of origin, study design) will be presented in tabular form. By analyzing the data, we will identify and categorize the performance factors identified as facilitators or barriers to the management of COVID-19 and situate them within the sub-concepts and dimensions of our conceptual framework. The relative frequency of each factor and sub-concept/dimension of performance will be reported. All results will be mapped and will follow the review’s conceptual framework.

### Review team

The principal investigator (JL) is a registered nurse and a post-doctoral fellow in the field of nursing sciences, who has expertise in IPC, performance analysis within a nursing organization, and evaluation of factors contributing to infection outbreaks. JL’s expertise is supported by the experience of several professors (ENT, DS, IB), who are experts in scoping review methodology within the field of nursing sciences. The review team also includes research professionals (EB, SR, KK) and a nursing student at the doctoral level in the field of nursing sciences (MJ), who are all proficient in searching databases, selecting articles, and preparing manuscripts.

### Consultation

In April 2021, two co-authors (JL, ENT) consulted two professors (DS, IB) with expertise in completing scoping reviews within the field of nursing sciences. These professors subsequently joined the review team to assist with the development of the search strategy.

## Discussion

### Implications

We will follow this protocol in completing our scoping review to identify and analyze the performance factors that have affected the management of the COVID-19 pandemic in LTCFs. The knowledge obtained through this review may assist with the development of more robust IPC measures to help prevent or mitigate future pandemics and infection outbreaks. An understanding of factors that have either facilitated or hindered the LTCF response to the COVID-19 pandemic can help enhance the quality of care for LTCF residents and ensure the safety of residents and staff both now and in the future.

### Limitations and strengths

Due to the evolving nature of the COVID-19 pandemic as well as the complexity of performing scoping reviews, the results of our review, when published, may not reflect the current public health profile. Furthermore, because this review focuses on the management of COVID-19, our results may not capture the indirect effects of the IPC measures implemented by LTCFs that have, nonetheless, had a direct influence on residents’ mental health, such as the restriction of group activities resulting in social isolation and loneliness [33, 34].

Despite these potential limitations, our scoping review will be the first to offer a comprehensive understanding of various factors affecting LTCF performance in the pandemic context using a multidimensional conceptual framework. The adopted framework of performance will guide us throughout the entire review process by providing a clear focus on the review’s aim and objectives. In addition to the conceptual framework, our team’s adherence to the JBI guidelines as a methodological framework will help ensure the rigor of our scoping review.

## Supplementary Information

**Additional file 1:** PRISMA-P.

**Additional file 2:** PRISMA-ScR.

**Additional file 3:** CINAHL.

**Additional file 4:** MEDLINE.

**Additional file 5: **CAIRN.

** Additional file 6:** Science Direct.

**Additional file 7:** Web of Science.

## Data Availability

Not applicable, as no datasets have been created during the production of this protocol.

## Abbreviations

DS: Drissa Sia
EB: Emilie Bélanger
ENT: Eric Nguemeleu Tchouaket
IPC: Infection prevention and control
IS: Idrissa Beogo
JBI: Joanna Briggs Institute
JL: Josiane Létourneau
KK: Katya Kruglova
LTCFs: Long-term care facilities
MJ: Maripier Jubinville
MSSS: Ministère de la Santé et des Services Sociaux du Québec
PRISMA-P: Preferred Reporting Items for Systematic Review and Meta-Analysis Protocols
PRISMA-ScR: Preferred Reporting Items for Systematic Reviews and Meta-Analyses Extension for Scoping Reviews
SR: Stephanie Robins
