# Supplementary material for: Identifying performance factors of long-term care facilities in the context of the COVID-19 pandemic: a scoping review protocol
Source: Syst Rev. 2022 Sep 23;11:203. doi: 10.1186/s13643-022-02069-1 (PMC9502645; doi:10.1186/s13643-022-02069-1)
Supplement: Supplementary file 3 — Additional file 3: CINAHL. [file 13643_2022_2069_MOESM3_ESM.docx]

**Supplementary File 3**

**CINAHL Search Strategy**

| Concept | Selected keywords |
| --- | --- |
| Efficiency | (MM "Organizational Efficiency") OR (MM "Productivity") OR (MM "Work Redesign") OR (MM "Public Health Administration") OR (MM "Organizational Development") OR (MM "Absenteeism") OR (MM "Impairment, Health Professional") OR (MM "Task Performance and Analysis") OR (MM "Vocational Guidance") OR (MM "Work-Life Balance") OR (MM "Employee Orientation") OR (MM "Mass Casualty Training") OR (MM "Behavioral Objectives") OR (MM "Curriculum") OR (MM "Education, Clinical") OR (MM "Education, Competency-Based") OR (MM "Education, Nonprofessional") OR (MM "Education, Health Sciences") OR (MM "Educational Measurement") OR (MM "Knowledge") OR (MM "Learning Environment") OR (MM "Learning Methods") OR (MM "Educational Technology") OR (MM "Educational Mobility") |
| OR |  |
| Effectiveness | (MM "Clinical Effectiveness") OR (MM "Cost Benefit Analysis") OR (MM "Economics, Organizations, Control") OR (MM “Economics”) OR (MM "Costs and Cost Analysis") OR (MM "Cost Control") OR (MM "Medical Savings Accounts") OR (MM "Health Care Costs") OR (MM "Therapeutic Index") |
| OR |  |
| Efficacy | (MM "Self-Efficacy") OR (MM "Personality Development") OR (MM "Confidence") OR (MM "Self-Actualization") OR (MM "Self-Awareness") OR (MM "Self Disclosure") OR (MM "Social Cognition") |
| OR |  |
| Security | (MM "Security Measures, Electronic") OR (MM "Security Measures") OR (MM "Data Security") OR (MM "National Labor Relations Board") OR (MM "United States Social Security Administration") OR (MM "Economic and Social Security") OR (MM "Food Assistance") OR (MM "Water Security") OR (MM "Data Breach") OR (MM "Security Enhancement (Iowa NIC)") OR (MM "Decision-Making Support (Iowa NIC)") OR (MM "Role Enhancement (Iowa NIC)") OR (MM "Self-Awareness Enhancement (Iowa NIC)") OR (MM "Support System Enhancement (Iowa NIC)") OR (MM "Food Security") OR (MM "Humanitarian Aid") OR (MM "Legislation, Drug") OR (MM "Legislation, Medical") OR (MM "Legislation, Nursing") OR (MM "Patient Protection and Affordable Care Act") OR (MM "Practice Acts") OR (MM "Emergency Medical Treatment and Active Labor Act") OR (MM "Clinical Laboratory Improvement Amendments") OR (MM "Health Insurance Portability and Accountability Act") OR (MM "Patient Self Determination Act") OR (MM "Legislation, Labor") |
| OR |  |
| Safety | (MM "Safety") OR (MM "Patient Safety") OR (MM "Accidents") OR (MM "Decontamination, Hazardous Materials") OR (MM "Disease Outbreaks") OR (MM "Disease Transmission") OR (MM "Drug Contamination") OR (MM "Environmental Microbiology") OR (MM "Environmental Pollution") OR (MM "Exposure to Violence") OR (MM "Equipment Contamination") OR (MM "Equipment Reuse") OR (MM "Fumigation") OR (MM "Food Safety") OR (MM "Hygiene") OR (MM "Infection Control") OR (MM "Mandatory Reporting") OR (MM "Mandatory Testing") OR (MM "Chemical Safety") OR (MM "Sanitation") OR (MM "Social Distancing") OR (MM "Voluntary Reporting") OR (MM "Occupational Safety") OR (MM "Equipment Safety") OR (MM "Fire Safety") OR (MM "Electrical Safety") OR (MM "Defibrillators") OR (MM "Equipment Alarm Systems") OR (MM "Equipment Design") OR (MM "Equipment Failure") OR (MM "Equipment Reliability") OR (MM "Firefighting Equipment and Supplies") OR (MM "Incontinence Aids") OR (MM "Intravenous Therapy Equipment and Supplies") OR (MM "Lifting and Transfer Equipment") OR (MM "Hypoallergenic Products") OR (MM "Safety-Net Providers") OR (MM "Safety Precautions (Saba CCC)") OR (MM "Environmental Safety (Saba CCC)") OR (MM "Equipment Safety (Saba CCC)") OR (MM "Individual Safety (Saba CCC)") OR (MM "Violence Control (Saba CCC)") OR (MM "Safety Component (Saba CCC)") OR (MM "Catheter Care") OR (MM "Cultural Safety") OR (MM "Emergency Care") OR (MM "Gerontologic Care") OR (MM "Night Care") OR (MM "Patient Handling") OR (MM "Seizure Precautions") OR (MM "Skin Care") OR (MM "Wound Care") OR (MM "Personal Protective Equipment") OR (MM "Protective Clothing") OR (MM "Respiratory Protective Devices") OR (MM "Smoke Alarms") OR (MM "Ear Protective Devices") OR (MM "Eye Protective Devices") OR (MM "Head Protective Devices") OR (MM "Hip Protectors") OR (MM "Masks") OR (MM "Mosquito Nets") OR (MM "Mouthguards") OR (MM "United States Occupational Safety and Health Administration") OR (MM "Emergency Care (Saba CCC)") OR (MM "Substance Abuse Control (Saba CCC)") OR (MM "Injury Risk (Saba CCC)") OR (MM "Violence Risk (Saba CCC)") OR (MM "Skin Integrity Component (Saba CCC)") |
| OR |  |
| Accessibility | (MM "Health Services Accessibility") OR (MM "Direct Access") OR (MM "Gatekeeping") OR (MM "Right to Health") OR (MM "Universal Health Care") OR (MM "Health Care Delivery") OR (MM "Healthcare Disparities") OR (MM "Health Care Delivery, Integrated") OR (MM "Health Care Reform") OR (MM "Learning Health System") OR (MM "Architectural Accessibility") OR (MM "Facility Design and Construction") OR (MM "Universal Design") OR (MM "Public Accommodation") OR (MM "Internet Access") OR (MM "Communication Barriers") OR (MM "Language") OR (MM "Nonverbal Communication") OR (MM "Social Networking") OR (MM "Neurolinguistic Programming") OR (MM "Conversation") OR (MM "Communication Skills") OR (MM "Patient Rights") OR (MM "Patient Access to Records") OR (MM "Right to Die") OR (MM "Right to Life") OR (MM "Treatment Refusal") OR (MM "Telehealth")) |
| OR |  |
| Equity | (MH "Prejudice") OR (MM "Ageism") OR (MM "Racism") OR (MM "Gender Equality") OR (MM "Racial Equality") OR (MM "Homophobia") OR (MM "Psychosocial Aspects of Illness") OR (MM "Sexism") OR (MM "Weight Bias") OR (MM "Family and Medical Leave") OR (MM "Pensions") OR (MM "Comparable Worth") OR (MM "Income") OR (MM "Socioeconomic Factors") OR (MM "Hospice and Palliative Nursing") |
| OR |  |
| Continuity | (MM "Continuity of Patient Care") OR (MM "Discharge Planning") OR (MM "Disease Management") OR (MM "Life Support Care") OR (MM "Multidisciplinary Care Team") OR (MM "Patient Centered Care") OR (MM "Patient Classification") OR (MM "Patient Rounds") OR (MM "Protocols") OR (MM "Primary Health Care") OR (MM "Clinical Conferences") OR (MM "Patient-Family Conferences") OR (MM "Patient Navigation") OR (MM "Age Specific Care") OR (MM "Case Management") OR (MM "Education, Nursing, Continuing") OR (MM "Continuing Education Providers") |
| OR |  |
| Adaptability | (MM "Acclimatization") OR (MM "Adaptation, Physiological") OR (MM "Aging") OR (MM "Recovery") OR (MM "Stress, Physiological") OR (MM "Human Needs (Physiology)") OR (MM "Homeostasis") OR (MM "Nutritional Physiology") OR (MM "Adaptation, Psychological") OR (MM "Adaptation, Occupational") OR (MM "Behavior") OR (MM "Defense Mechanisms") OR (MM "Emotions") OR (MM "Human Development") OR (MM "Motivation") OR (MM "Personal Values") OR (MM "Psychosocial Functioning") OR (MM "Physical Education and Training") OR (MM "Physical Education, Adapted") OR (MM "Psychosocial Adaptation (Iowa NOC)") OR (MM "Psychosocial Health (Iowa NOC)") OR (MM "Psychological Well-Being (Iowa NOC)") OR (MM "Acceptance: Health Status (Iowa NOC)") OR (MM "Coping (Iowa NOC)") OR (MM "Grief Resolution (Iowa NOC)") OR (MM "Psychosocial Adjustment: Life Change (Iowa NOC)") OR OR (MM "Social Interaction (Iowa NOC)") OR (MM "Caregiver Adaptation to Patient Institutionalization (Iowa NOC)") OR (MM "Family Caregiver Status (Iowa NOC)") OR (MM "Caregiver-Patient Relationship (Iowa NOC)") OR (MM "Caregiver Performance: Direct Care (Iowa NOC)") OR (MM "Caregiver Performance: Indirect Care (Iowa NOC)") OR (MM "Caregiver Stressors (Iowa NOC)") OR (MH "Caregiving Endurance Potential (Iowa NOC)") OR (MM "Caregiver Lifestyle Disruption (Iowa NOC)") OR (MM "Family Health (Iowa NOC)") |
| OR |  |
| Satisfaction | (MM "Job Satisfaction") OR (MM "Stress, Occupational") OR (MM "Presenteeism") OR (MM "Professional Recognition") OR (MM "Professional Image") OR (MM "Patient Satisfaction") OR (MM "Patient Preference") OR (MM "Patient Compliance") OR (MM "Health Beliefs") OR (MM "Attitude to Health") OR OR (MM "Attitude to Vaccines") OR (MM "Attitude to Medical Treatment") OR (MM "Attitude of Health Personnel") OR (MH "Attitude to Aging") OR (MM "Attitude to Change") OR (MH "Attitude to Death") OR (MM "Attitude to Illness") OR (MM "Attitude to Disability") OR (MM "Attitude to Risk") OR (MM "Employee Attitudes") OR (MM "Family Attitudes") OR (MM "Patient Attitudes") OR (MH "Personal Satisfaction")) |
| OR |  |
| Resource Management | (MM "Personnel Management") OR (MH "Conflict Management") OR (MM "Cross Training") OR (MM "Delegation of Authority") OR (MM "Employee Discipline") OR (MM "Employee Grievances") OR (MM "Employee Incentive Programs") OR (MM "Employee Performance Appraisal") OR (MM "Employee Rights") OR (MM "Employer-Employee Relations") OR (MM "Employment Termination") OR (MM "Exchange Programs") OR (MM "Job Characteristics") OR (MM "Job Description") OR (MM "Job Experience") OR (MM "Job Performance") OR (MM "Job Re-Entry") OR (MM "Mentorship") OR (MM "Nursing Manpower") OR (MM "Personnel Loyalty") OR (MM "Personnel Recruitment") OR (MM "Personnel Selection") OR (MM "Personnel Shortage") OR (MM "Personnel Staffing and Scheduling") OR (MM "Personnel Turnover") OR (MM "Physician Incentive Plans") OR (MM "Placement Agencies") OR (MM "Promotion and Tenure") OR (MM "Salaries and Fringe Benefits") OR (MM "Staff Development") OR (MM "Supervisors and Supervision") OR (MM "Team Building") OR (MM "Teamwork") OR (MM "Work Engagement") OR (MM "Workload") OR (MM "Professional Discipline") OR (MM "Professional Practice") OR (MM "Public Relations") OR (MM "Quality of Working Life") OR (MM "Rules and Regulations") OR (MM "Shared Governance") OR (MM "Time Management") OR (MM "Management Information Systems") OR (MM "Appointment and Scheduling Information Systems") OR (MM "Decision Support Systems, Management") OR (MM "Financial Information Systems") OR (MM "Personnel Staffing and Scheduling Information Systems") OR (MM "Practice Management Information Systems") OR (MM "Domain II: Psychosocial Domain (Omaha)") OR (MM "Abuse (Omaha)") OR (MM "Grief (Omaha)") OR (MM "Growth and Development (Omaha)") OR (MM "Interpersonal Relationships (Omaha)") OR (MM "Mental Health (Omaha)") OR (MM "Neglect (Omaha)") OR (MM "Management") OR (MM "Appointments and Schedules") OR (MM "Goals and Objectives") OR (MM "Documentation") OR (MM "Governing Board") OR (MM "Information Management") OR (MM "Management Styles") OR (MM "Negotiation") OR (MM "Networking, Professional") OR (MM "Organizational Compliance") OR (MM "Organizational Development") OR (MM "Organizational Efficiency") OR (MM "Organizational Policies") OR (MM "Patient Identification") OR (MM "Quality Assurance") |
| OR |  |
| Resource Mobilization | (MM "Health Resource Utilization") OR (MM "Health Facility Planning") OR (MM "Health Initiative 2000") OR (MM "Health Priorities") OR (MM "Health Resource Allocation") OR (MM "Health Services Needs and Demand") OR (MM "Strategic Planning") OR (MM "National Health Programs") OR (MM "State Health Plans") OR (MM "Information Resources") OR (MM "Electronic Publications") OR (MM "Health Information") OR (MM "Print Materials") OR (MM "Reference Tools") OR (MM "Teaching Materials") OR (MM "Mail") OR (MM "Telecommunications") OR (MM "Relocation") OR (MM "Knowledge: Health Resources (Iowa NOC)") OR (MM "Knowledge: Infection Control (Iowa NOC)") OR (MM "Knowledge: Personal Safety (Iowa NOC)") OR (MM "Knowledge: Treatment Procedures (Iowa NOC)") OR (MM "Health Belief (Iowa NOC)") OR (MM "Health Beliefs: Perceived Resources (Iowa NOC)") OR (MM "Health Beliefs: Perceived Control (Iowa NOC)") OR (MM "Health Beliefs: Perceived Ability to Perform (Iowa NOC)") OR (MM "Health Beliefs: Perceived Threat (Iowa NOC)") OR (MM "Communication with Community Resources (Omaha)") OR (MM "Facilities, Manpower and Services") |
| OR |  |
| Structures | (MH "Organizational Structure") OR (MH "Health Services Administration") OR (MM "Evaluation**"**) OR (MM "Knowledge Management") OR (MH "Medication Systems") OR (MH "Multiinstitutional Systems") OR (MH "Organizational Change") OR (MH "Organizational Culture") OR (MM "Pharmacy Administration") OR (MM "Planning Techniques") OR (**MM "**Product Acquisition**")** OR (MM "Program Development") OR (MH "Risk Management") OR (MH "Shared Services, Health Care") OR (MM "Quality Management, Organizational") OR (MM "Product Line Management") OR (MM "Organizational Restructuring") OR (MM "Nursing Administration") OR (MM "Materials Management") OR (MM "Health Facility Administration") OR (MM "Clinical Governance") |
| OR |  |
| Care Procedures | (MM "Charting") OR (MM "Coding") OR (MM "Death Certificates") OR (MM "Diaries") OR (MM "Employee Records") OR (MH "Medical Orders") OR (MM "Medical Records") OR (MM "Medical Transcription") OR (MM "Nursing Orders") OR (MM "Organ Donor Cards") OR (MM "Policy and Procedure Manuals") OR (MM "Shift Reports") OR (MM "Health Services Misuse") OR (MM "Unnecessary Procedures") OR (MM "Device Removal") OR (MM "Drainage") OR (MM "Minimally Invasive Procedures") OR (MM "Bedmaking") OR (MM "Bowel Preparation") OR (MM "Catheterization") OR (MM "Catheter Placement Determination") OR (MM "Diet") OR (MM "Insertion Sites") OR (MM "Endovascular Procedures") OR (MM "Patient Positioning") OR (MM "Perfusion") OR (MM "Physical Stimulation") OR (MM "Product Labeling") OR (MM "Prosthetic Fitting") OR (MM "Substance Withdrawal, Controlled") OR (MM "Therapeutic Irrigation") OR (MM "Product Evaluation") OR (MM "Body Weights and Measures") OR (MM "Bandaging Techniques") OR (MM "Immobilization") OR (MM "Noninvasive Procedures") OR (MM "Practice Guidelines") OR (MM "Therapeutic Index") OR (MM "Patient Care") OR (MM "Nursing Process") OR (“Nursing Care”) OR (MM "Nursing Assessment") OR (MM "Nursing Care Plans") OR (MM "Nursing Diagnosis") OR (MM "Nursing Interventions") |
| OR |  |
| Results | (MM "Outcome Assessment") OR (MM "Quality of Health Care") OR (MM "Outcomes (Health Care)") OR (MM "Medical Futility") OR (MM "Nursing Outcomes") OR (MM "Patient-Reported Outcomes") OR (MM "Treatment Outcomes") OR (MM "Quality Assurance") OR (MM "Quality Assessment") OR (MM "Clinical Documentation Improvement") OR (MM "Clinical Indicators") OR (MM "Health Plan Employer Data and Information Set") OR (MM "Joint Commission Core Measures") OR (MM "Nursing Audit") OR (MM "Outcome Assessment Information Set") OR (MM "Peer Review") OR (MM "Process Assessment (Health Care)") OR (MM "Utilization Review") OR (MM "Program Evaluation") OR (MM "Accountability") OR (MM "Guideline Adherence") OR (MM "Meaningful Use") OR (MM "Professional Compliance") OR (MM **"**Public Reporting of Healthcare Data**")** OR (MM "Quality of Nursing Care") OR (MM "Drug Efficacy") OR (MM "Fatal Outcome") OR (MM "Treatment Failure") OR (MM "Prognosis") OR (MM "Treatment Termination") OR (MM "Miscellaneous Techniques") OR (MM "Global Burden of Disease") OR (MM "Health Services Research") OR (MM "Outcomes Research") OR (MM "Quality of Care Research") OR (MM "Audit") |
| AND |  |
| Long term care | (MM "Long Term Care") OR "long term care" OR (MM "Nursing Home Patients") OR (MM "Hospice Patients") OR TI (‘Long-Term Care' or 'Assisted-Living Facilities' or 'long-term-care facility' or 'Homes for the Aged' or 'Nursing Homes' or 'nursing home' or 'long-term care' or 'retirement home' or ‘hospice patients’ or ‘hospice care’) OR AB ( 'Long-Term Care' or 'Assisted-Living Facilities' or 'long-term-care facility' or 'Homes for the Aged' or 'Nursing Homes' or 'nursing home' or 'long-term care' or 'retirement home' or ‘hospice patients’ or ‘hospice care’) |
| AND |  |
| COVID-19 | TI (covid-19 or coronavirus or 2019-ncov or sars-cov-2 or cov-19 or covid) OR AB (covid-19 or coronavirus or 2019-ncov or sars-cov-2 or cov-19 or covid) |
