# Supplementary material for: Identifying performance factors of long-term care facilities in the context of the COVID-19 pandemic: a scoping review protocol
Source: Syst Rev. 2022 Sep 23;11:203. doi: 10.1186/s13643-022-02069-1 (PMC9502645; doi:10.1186/s13643-022-02069-1)
Supplement: Supplementary file 4 — Additional file 4: MEDLINE. [file 13643_2022_2069_MOESM4_ESM.docx]

**Supplementary File 4**

**MEDLINE Search Strategy**

| **Performance** |
| --- |
| ***Efficiency*** Capacité d’utiliser les ressources disponibles (humaines, matérielles, financières, technologiques et informationnelles) de façon optimale  (MM "Efficiency") OR (MM "Records") OR (MM "Professional Autonomy") OR (MM "Multi-Institutional Systems") OR (MM "Medication Systems") OR (MM "Program Development") OR (MM "Public Relations") OR (MM "Public Health Administration") OR (MM "Efficiency, Organizational") OR (MM "Resource Allocation") OR TI ( (Efficiency) OR (Professional Autonomy) OR (Records) OR (Multi-Institutional Systems) OR (Medication Systems) OR (Program Development) OR (Public Relations) OR (Public Health Administration) OR (Efficiency, Organizational) OR (Resource Allocation)) OR AB ( (Efficiency) OR (Professional Autonomy) OR (Records) OR (Multi-Institutional Systems) OR (Medication Systems) OR (Program Development) OR (Public Relations) OR (Public Health Administration) OR (Efficiency, Organizational) OR (Resource Allocation))  **OR**  ***Effectiveness***  (MM "Cost Sharing") OR (MM "Cost of Illness") OR (MM "Cost-Benefit Analysis") OR (MM "Cost Control") OR (MM "Cost Allocation") OR (MH "Costs and Cost Analysis") OR (MM "Prognosis") OR (MM "Treatment Failure") OR (MM "Medical Futility") OR (MM "Diagnosis") OR (MM "Program Evaluation") OR (MH "Product Surveillance, Postmarketing") OR (MM "Drug Evaluation") OR (MM "Investigative Techniques") OR (MM "Quality Indicators, Health Care") OR (MM "Utilization Review") OR (MM "Quality Assurance, Health Care") OR (MM "Professional Review Organizations") OR (MM "Peer Review, Health Care") OR (MM "Clinical Competence") OR (MH "Guideline Adherence") OR (MM "Advance Directive Adherence") OR (MM "Independent Medical Evaluation") OR (MM "Data Collection") OR TI ((Cost Sharing) OR (Cost of Illness) OR (Cost-Benefit Analysis) OR (Cost Control) OR (Cost Allocation) OR (Costs and Cost Analysis) OR (Prognosis) OR (Treatment Failure) OR (Medical Futility) OR (Diagnosis) OR (Program Evaluation) OR (Product Surveillance, Postmarketing) OR (Drug Evaluation) OR (Investigative Techniques) OR (Quality Indicators, Health Care) OR (Utilization Review) OR (Quality Assurance, Health Care) OR (Professional Review Organizations) OR (Peer Review, Health Care) OR (Clinical Competence) OR (Guideline Adherence) OR (Advance Directive Adherence) OR (Independent Medical Evaluation) OR (Data Collection)) OR AB ((Cost Sharing) OR (Cost of Illness) OR (Cost-Benefit Analysis) OR (Cost Control) OR (Cost Allocation) OR (Costs and Cost Analysis) OR (Prognosis) OR (Treatment Failure) OR (Medical Futility) OR (Diagnosis) OR (Program Evaluation) OR (Product Surveillance, Postmarketing) OR (Drug Evaluation) OR (Investigative Techniques) OR (Quality Indicators, Health Care) OR (Utilization Review) OR (Quality Assurance, Health Care) OR (Professional Review Organizations) OR (Peer Review, Health Care) OR (Clinical Competence) OR (Guideline Adherence) OR (Advance Directive Adherence) OR (Independent Medical Evaluation) OR (Data Collection))  **OR**  ***Efficacy***  (MM "Self Efficacy") OR (MM "Therapeutic Index") OR (MM "Treatment Outcome") OR (MM "Outcome Assessment, Health Care") OR (MM "Patient Outcome Assessment") OR (MM "Outcome and Process Assessment, Health Care") OR (MM "Quality of Health Care") OR (MM "Health Care Evaluation Mechanisms") OR (MM "Technology Assessment, Biomedical") OR (MM "Health Planning") OR (MM "Neurological Rehabilitation") OR (MM "Health Services") OR (MM "Rehabilitation, Vocational") OR (MM "Product Surveillance, Postmarketing") OR (MM "Prescription Drug Monitoring Programs") OR (MM "Pharmacovigilance") OR (MM "Adverse Drug Reaction Reporting Systems") OR TI ( (Self Efficacy) OR (Therapeutic Index) OR (Treatment Outcome) OR (Outcome Assessment, Health Care) OR (Patient Outcome Assessment) OR (Outcome and Process Assessment, Health Care) OR (Quality of Health Care) OR (Health Care Evaluation Mechanisms) OR (Technology Assessment, Biomedical) OR (Health Planning) OR (Activities of Daily Living) OR (Neurological Rehabilitation) OR (Health Services) OR (Rehabilitation, Vocational) OR (Product Surveillance, Postmarketing) OR (Prescription Drug Monitoring Programs) OR (Pharmacovigilance) OR (Adverse Drug Reaction Reporting Systems)) OR AB ( (Self Efficacy) OR (Therapeutic Index) OR (Treatment Outcome) OR (Outcome Assessment, Health Care) OR (Patient Outcome Assessment) OR (Outcome and Process Assessment, Health Care) OR (Quality of Health Care) OR (Health Care Evaluation Mechanisms) OR (Technology Assessment, Biomedical) OR (Health Planning) OR (Activities of Daily Living) OR (Neurological Rehabilitation) OR (Health Services) OR (Rehabilitation, Vocational) OR (Product Surveillance, Postmarketing) OR (Prescription Drug Monitoring Programs) OR (Pharmacovigilance) OR (Adverse Drug Reaction Reporting Systems))  **OR**  ***Security***  Capacité de minimiser les risques associés aux interventions et aux milieux de soins et de services pour les résidents (incluant le personnel) Surveillance, Organisation des lieux physiques et d’ingénierie, Mesures de protection individuelles, implantation des pratiques exemplaires, etc.)  (MM "Food Security") OR (MM "Food Supply") OR (MM "Pensions") OR (MM "Health Benefit Plans, Employee") OR (MM "Salaries and Fringe Benefits") OR (MM "Legislation, Medical") OR (MM "Privacy") OR (MM "Legislation, Food") OR (MM "Legislation, Dental") OR (MM "Legislation, Pharmacy") OR (MM "Legislation, Nursing") OR (MM "Employee Retirement Income Security Act") OR (MM "Security Measures") OR (MM "Computer Security") OR (MM "Biometric Identification") OR (MM "Patient Identification Systems") OR (MM "Old Age Assistance") OR (MM "Social Security") OR (MM "Insurance, Disability") OR (MM "Insurance Coverage") OR (MM "Risk Reduction Behavior") OR TI ( (Food Security) OR (Food Supply) OR (Pensions) OR (Health Benefit Plans, Employee) OR (Salaries and Fringe Benefits) OR (Legislation, Medical) OR (Privacy) OR (Legislation, Food) OR (Legislation, Dental) OR (Legislation, Pharmacy) OR (Legislation, Nursing) OR (Employee Retirement Income Security Act) OR (Security Measures) OR (Computer Security) OR (Biometric Identification) OR (Patient Identification Systems) OR (Old Age Assistance) OR (Social Security) OR (Insurance, Disability) OR (Insurance Coverage) OR (Risk Reduction Behavior)) OR AB ( (Food Security) OR (Food Supply) OR (Pensions) OR (Health Benefit Plans, Employee) OR (Salaries and Fringe Benefits) OR (Legislation, Medical) OR (Privacy) OR (Legislation, Food) OR (Legislation, Dental) OR (Legislation, Pharmacy) OR (Legislation, Nursing) OR (Employee Retirement Income Security Act) OR (Security Measures) OR (Computer Security) OR (Biometric Identification) OR (Patient Identification Systems) OR (Old Age Assistance) OR (Social Security) OR (Insurance, Disability) OR (Insurance Coverage) OR (Risk Reduction Behavior))  **OR**  ***Safety***  (MM "Safety") OR (MM "Chemical Safety") OR (MM "Accident Prevention") OR (MM "Safety Management") OR (MM "Patient Harm") OR (MM "Hazard Analysis and Critical Control Points") OR (MM "Containment of Biohazards") OR (MM "Accidents") OR (MM "Environment and Public Health") OR (MM "Patient Safety") OR (MM "Equipment Safety") OR (MM "Immobilization") OR (MM "Equipment Reuse") OR (MM "Equipment Failure") OR (MM "Equipment Design") OR (MM "Drug Development") OR (MM "Toxicity Tests") OR (MM "Substance Abuse Detection") OR (MM "Food Safety") OR (MM "Food Inspection") OR (MM "Food Contamination") OR (MM "Food Quality") OR (MM "Safety-net Providers") OR (MM "Patient Care Management") OR (MM "Sanitation") OR (MM "Waste Management") OR (MM "Water Purification") OR (MM "Waste Disposal Facilities") OR (MM "Waste Disposal, Fluid") OR (MM "Toilet Facilities") OR (MM "Sanitary Engineering") OR (MM "Refuse Disposal") OR (MM "Drainage, Sanitary") OR (MM "Hygiene") OR (MM "Equipment Contamination") OR (MM "Epidemiologic Factors") OR (MM "Environmental Pollution") OR (MM "Environmental Microbiology") OR (MM "Drug Contamination") OR (MM "Disease Transmission, Infectious") OR (MM "Disease Outbreaks") OR (MM "Disease Eradication") OR (MM "Product Recalls and Withdrawals") OR (MM "Device Approval") OR (MM "Public Health Practice") OR (MM "Consumer Product Safety") OR (MM "Drug and Narcotic Control") OR (MM "Legislation, Drug") OR (MM "Drug Approval") OR (MM "Drug Recalls") OR (MM "Safety-Based Medical Device Withdrawals") OR (MM "Safety-Based Drug Withdrawals") OR (MH "Medical Device Recalls") OR (MM "Risk Management") OR (MM "Material Safety Data Sheets") OR (MM "United States Occupational Safety and Health Administration") OR (MH "National Institute for Occupational Safety and Health, U.S.") OR TI ( (Safety) OR (Chemical Safety) OR (Accident Prevention) OR (Safety Management) OR (Patient Harm) OR (Hazard Analysis and Critical Control Points) OR (Containment of Biohazards) OR (Accidents) OR (Environment and Public Health) OR (Patient Safety) OR (Equipment Safety) OR (Immobilization) OR (Equipment Reuse) OR (Equipment Failure) OR (Equipment Design) OR (Drug Development) OR (Toxicity Tests) OR (Substance Abuse Detection) OR (Food Safety) OR (Food Inspection) OR (Food Contamination) OR (Food Quality) OR (Safety-net Providers) OR (Patient Care Management) OR (Sanitation) OR (Waste Management) OR (Water Purification) OR (Waste Disposal Facilities) OR (Waste Disposal, Fluid) OR (Toilet Facilities) OR (Sanitary Engineering) OR (Refuse Disposal) OR (Drainage, Sanitary) OR (Hygiene) OR (Equipment Contamination) OR (Epidemiologic Factors) OR (Environmental Pollution) OR (Environmental Microbiology) OR (Drug Contamination) OR (Disease Transmission, Infectious) OR (Disease Outbreaks) OR (Disease Eradication) OR (Product Recalls and Withdrawals) OR (Device Approval) OR (Public Health Practice) OR (Consumer Product Safety) OR (Drug and Narcotic Control) OR (Legislation, Drug) OR (Drug Approval) OR (Drug Recalls) OR (Safety-Based Medical Device Withdrawals) OR (Safety-Based Drug Withdrawals) OR (Medical Device Recalls) OR (Risk Management) OR (Material Safety Data Sheets) OR (United States Occupational Safety and Health Administration) OR (National Institute for Occupational Safety and Health, U.S.) ) OR AB ( (Safety) OR (Chemical Safety) OR (Accident Prevention) OR (Safety Management) OR (Patient Harm) OR (Hazard Analysis and Critical Control Points) OR (Containment of Biohazards) OR (Accidents) OR (Environment and Public Health) OR (Patient Safety) OR (Equipment Safety) OR (Immobilization) OR (Equipment Reuse) OR (Equipment Failure) OR (Equipment Design) OR (Drug Development) OR (Toxicity Tests) OR (Substance Abuse Detection) OR (Food Safety) OR (Food Inspection) OR (Food Contamination) OR (Food Quality) OR (Safety-net Providers) OR (Patient Care Management) OR (Sanitation) OR (Waste Management) OR (Water Purification) OR (Waste Disposal Facilities) OR (Waste Disposal, Fluid) OR (Toilet Facilities) OR (Sanitary Engineering) OR (Refuse Disposal) OR (Drainage, Sanitary) OR (Hygiene) OR (Equipment Contamination) OR (Epidemiologic Factors) OR (Environmental Pollution) OR (Environmental Microbiology) OR (Drug Contamination) OR (Disease Transmission, Infectious) OR (Disease Outbreaks) OR (Disease Eradication) OR (Product Recalls and Withdrawals) OR (Device Approval) OR (Public Health Practice) OR (Consumer Product Safety) OR (Drug and Narcotic Control) OR (Legislation, Drug) OR (Drug Approval) OR (Drug Recalls) OR (Safety-Based Medical Device Withdrawals) OR (Safety-Based Drug Withdrawals) OR (Medical Device Recalls) OR (Risk Management) OR (Material Safety Data Sheets) OR (United States Occupational Safety and Health Administration) OR (National Institute for Occupational Safety and Health, U.S.) )  **OR**  ***Accessibility***  Capacité de fournir les soins et les services requis, à l’endroit et au moment opportun  (MM "Activities of Daily Living") OR (MM "Architectural Accessibility") OR (MM "Facility Design and Construction") OR (MM "Interior Design and Furnishings") OR (MM "Hospital Design and Construction") OR (MM "Floors and Floorcoverings") OR (MM "Elevators and Escalators") OR (MM "Health Services Accessibility") OR (MM "Universal Health Care") OR (MM "Health Care Reform") OR (MM "Delivery of Health Care, Integrated") OR (MM "After-Hours Care") OR (MM "Delivery of Health Care") OR (MM "Telemedicine") OR (MH "Culturally Competent Care") OR (MM "Managed Care Programs") OR (MM "Learning Health System") OR (MM "Practice Patterns, Pharmacists'") OR (MM "Practice Patterns, Physicians'") OR (MM "Practice Patterns, Nurses'") OR (MM "Practice Patterns, Dentists'") OR (MM "Health Services Administration") OR (MM "Product Line Management") OR (MM "Professional-Patient Relations") OR (MM "Nurses Improving Care for Health System Elders") OR (MM "Needs Assessment") OR (MM "Health Facility Environment") OR (MM "Health Services Needs and Demand") OR (MM "Health Facility Size") OR (MM "Health Resources") OR (MM "Health Care Rationing") OR (MM "Health Care Costs") OR (MM "Health Priorities") OR (MM "Health Expenditures") OR (MM "Attitude to Health") OR (MM "Attitude to Death") OR (MM "Attitude of Health Personnel") OR (MM "Health Care Quality, Access, and Evaluation") OR (MM "Internet Access") OR (MM "Social Networking") OR (MM "Internet Use") OR (MM "Nonverbal Communication") OR (MM "Information Literacy") OR (MM "Information Dissemination") OR (MM "Health Communication") OR (MM "Diffusion of Innovation") OR (MM "Communication Barriers") OR (MM "Access to Information") OR (MM "Communication") OR (MM "Information Seeking Behavior") OR (MM "Computer Literacy") OR (MM "Reminder Systems") OR (MM "Internet-Based Intervention") OR (MM "Internet") OR (MM "Computer Communication Networks") OR (MM "Social Media") OR (MM "Computer Systems") OR (MM "Equipment and Supplies") OR (MM "Patient Access to Records") OR (MM "Social Control, Formal") OR (MM "Treatment Refusal") OR (MM "Informed Consent") OR (MH "Confidentiality") OR (MM "Medicare") OR (MM "Medical Assistance") OR (MM "Public Assistance") OR (MM "Financing, Government") OR (MM "Financing, Organized") OR (MM "Economics") OR (MM "Health Care Economics and Organizations") OR (MM "Insurance, Health") OR (MM "Insurance") OR (MM "Legislation as Topic") OR (MM "Medicare Access and CHIP Reauthorization Act of 2015") OR TI ( (Activities of Daily Living) (Architectural Accessibility) OR (Facility Design and Construction) OR (Interior Design and Furnishings) OR (Hospital Design and Construction) OR (Floors and Floorcoverings) OR (Elevators and Escalators) OR (Health Services Accessibility) OR (Universal Health Care) OR (Health Care Reform) OR (Delivery of Health Care, Integrated) OR (After-Hours Care) OR (Delivery of Health Care) OR (Telemedicine) OR (Culturally Competent Care) OR (Managed Care Programs) OR (Learning Health System) OR (Practice Patterns, Pharmacists) OR (Practice Patterns, Physicians) OR (Practice Patterns, Nurses) OR (Practice Patterns, Dentists) OR (Health Services Administration) OR (Product Line Management) OR (Professional-Patient Relations) OR (Nurses Improving Care for Health System Elders) OR (Needs Assessment) OR (Health Facility Environment) OR (Health Services Needs and Demand) OR (Health Facility Size) OR (Health Resources) OR (Health Care Rationing) OR (Health Care Costs) OR (Health Priorities) OR (Health Expenditures) OR (Attitude to Health) OR (Attitude to Death) OR (Attitude of Health Personnel) OR (Health Care Quality, Access, and Evaluation) OR (Internet Access) OR (Social Networking) OR (Internet Use) OR (Nonverbal Communication) OR (Information Literacy) OR (Information Dissemination) OR (Health Communication) OR (Diffusion of Innovation) OR (Communication Barriers) OR (Access to Information) OR (Communication) OR (Information Seeking Behavior) OR (Computer Literacy) OR (Reminder Systems) OR (Internet-Based Intervention) OR (Internet) OR (Computer Communication Networks) OR (Social Media) OR (Computer Systems) OR (Equipment and Supplies) OR (Patient Access to Records) OR (Civil Rights) OR (Social Control, Formal) OR (Treatment Refusal) OR (Informed Consent) OR (Confidentiality) OR (Medicare) OR (Medical Assistance) OR (Public Assistance) OR (Financing, Government) OR (Financing, Organized) OR (Economics) OR (Health Care Economics and Organizations) OR (Insurance, Health) OR (Insurance) OR (Legislation as Topic) OR (Medicare Access and CHIP Reauthorization Act of 2015) ) OR AB ( (Activities of Daily Living) OR (Architectural Accessibility) OR (Facility Design and Construction) OR (Interior Design and Furnishings) OR (Hospital Design and Construction) OR (Floors and Floorcoverings) OR (Elevators and Escalators) OR (Health Services Accessibility) OR (Universal Health Care) OR (Health Care Reform) OR (Delivery of Health Care, Integrated) OR (After-Hours Care) OR (Delivery of Health Care) OR (Telemedicine) OR (Culturally Competent Care) OR (Managed Care Programs) OR (Learning Health System) OR (Practice Patterns, Pharmacists) OR (Practice Patterns, Physicians) OR (Practice Patterns, Nurses) OR (Practice Patterns, Dentists) OR (Health Services Administration) OR (Product Line Management) OR (Professional-Patient Relations) OR (Nurses Improving Care for Health System Elders) OR (Needs Assessment) OR (Health Facility Environment) OR (Health Services Needs and Demand) OR (Health Facility Size) OR (Health Resources) OR (Health Care Rationing) OR (Health Care Costs) OR (Health Priorities) OR (Health Expenditures) OR (Attitude to Health) OR (Attitude to Death) OR (Attitude of Health Personnel) OR (Health Care Quality, Access, and Evaluation) OR (Internet Access) OR (Social Networking) OR (Internet Use) OR (Nonverbal Communication) OR (Information Literacy) OR (Information Dissemination) OR (Health Communication) OR (Diffusion of Innovation) OR (Communication Barriers) OR (Access to Information) OR (Communication) OR (Information Seeking Behavior) OR (Computer Literacy) OR (Reminder Systems) OR (Internet-Based Intervention) OR (Internet) OR (Computer Communication Networks) OR (Social Media) OR (Computer Systems) OR (Equipment and Supplies) OR (Patient Access to Records) OR (Civil Rights) OR (Social Control, Formal) OR (Treatment Refusal) OR (Informed Consent) OR (Confidentiality) OR (Medicare) OR (Medical Assistance) OR (Public Assistance) OR (Financing, Government) OR (Financing, Organized) OR (Economics) OR (Health Care Economics and Organizations) OR (Insurance, Health) OR (Insurance) OR (Legislation as Topic) OR (Medicare Access and CHIP Reauthorization Act of 2015) )  **OR**  ***Equity*** Capacité de fournir les soins et les services en fonction des besoins et sans égard aux caractéristiques personnelles non reliées aux besoins, comme le revenu, l’éducation, le lieu de résidence, etc  (MM "Healthcare Disparities") OR (MM "Human Rights") OR (MM "Civil Rights") OR (MM "Patient Rights") OR (MM "Right to Die") OR (MM "Gender Equity") OR (MM "Xenophobia") OR (MM "Weight Prejudice") OR (MM "Sexism") OR (MM "Racism") OR (MM "Homophobia") OR (MM "Ageism") OR (MM "Prejudice") OR (MM "Social Behavior") OR (MM "Sociological Factors") OR (MM "Social Problems") OR (MM "Social Work") OR (MM "Health Equity") OR (MM "Right to Health") OR (MM "Sick Leave") OR (MM "Family Leave") OR (MM "Remuneration") OR (MM "Income") OR (MM "Population Characteristics") OR (MM "Negotiating") OR (MM "Work Engagement") OR (MM "Strikes, Employee") OR (MM "Staff Development") OR (MM "Employee Performance Appraisal") OR (MM "Collective Bargaining") OR (MM "Physician Incentive Plans") OR (MM "Patient Credit and Collection") OR (MM "Financial Audit") OR (MM "Financial Statements") OR (MM "Financial Management") OR (MM "Accounting") OR (MM "Tax Equity and Fiscal Responsibility Act") OR (MM "Patient Protection and Affordable Care Act") OR (MM "Patient Self-Determination Act") OR (MM "Patient Freedom of Choice Laws") OR (MM "Medicaid") OR TI ( (Healthcare Disparities) OR (Human Rights) OR (Civil Rights) OR (Patient Rights) OR (Right to Die) OR (Gender Equity) OR (Xenophobia) OR (Weight Prejudice) OR (Sexism) OR (Racism) OR (Homophobia) OR (Ageism) OR (Prejudice) OR (Social Behavior) OR (Sociological Factors) OR (Social Problems) OR (Social Work) OR (Health Equity) OR (Right to Health) OR (Sick Leave) OR (Family Leave) OR (Remuneration) OR (Income) OR (Population Characteristics) OR (Negotiating) OR (Work Engagement) OR (Strikes, Employee) OR (Staff Development) OR (Employee Performance Appraisal) OR (Collective Bargaining) OR (Physician Incentive Plans) OR (Patient Credit and Collection) OR (Financial Audit) OR (Financial Statements) OR (Financial Management) OR (Accounting) OR (Tax Equity and Fiscal Responsibility Act) OR (Patient Protection and Affordable Care Act) OR (Patient Self-Determination Act) OR (Patient Freedom of Choice Laws) OR (Medicaid) ) OR AB ( (Healthcare Disparities) OR (Human Rights) OR (Civil Rights) OR (Patient Rights) OR (Right to Die) OR (Gender Equity) OR (Xenophobia) OR (Weight Prejudice) OR (Sexism) OR (Racism) OR (Homophobia) OR (Ageism) OR (Prejudice) OR (Social Behavior) OR (Sociological Factors) OR (Social Problems) OR (Social Work) OR (Health Equity) OR (Right to Health) OR (Sick Leave) OR (Family Leave) OR (Remuneration) OR (Income) OR (Population Characteristics) OR (Negotiating) OR (Work Engagement) OR (Strikes, Employee) OR (Staff Development) OR (Employee Performance Appraisal) OR (Employee Incentive Plans) OR (Employee Grievances) OR (Collective Bargaining) OR (Physician Incentive Plans) OR (Patient Credit and Collection) OR (Financial Audit) OR (Financial Statements) OR (Financial Management) OR (Accounting) OR (Tax Equity and Fiscal Responsibility Act) OR (Patient Protection and Affordable Care Act) OR (Patient Self-Determination Act) OR (Patient Freedom of Choice Laws) OR (Medicaid) )  **OR**  ***Continuity*** Capacité de fournir les soins et les services requis, de façon intégrée et coordonnée. Dans le contexte de services organisés par niveau (services de première ligne, deuxième ligne (services spécialisés), troisième ligne (services surspécialisés)), la continuité doit s’opérer à l’intérieur d’un niveau et entre les niveaux  (MM "Continuity of Patient Care") OR (MM "Patient Care") OR (MM "Patient Transfer") OR (MM "Withholding Treatment") OR (MM "Transitional Care") OR (MM "Time-to-Treatment") OR (MM "Terminal Care") OR (MM "Subacute Care") OR (MM "Preoperative Care") OR (MM "Patient Positioning") OR (MM "Palliative Care") OR (MM "Nursing Care") OR (MM "Night Care") OR (MM "Life Support Care") OR (MM "Institutionalization") OR (MM "Hospitalization") OR (MM "Critical Care") OR (MM "Episode of Care") OR (MM "Duration of Therapy") OR (MM "Delayed Diagnosis") OR (MM "Custodial Care") OR (MM "Retention in Care") OR (MM "Patient Handoff") OR (MM "Aftercare") OR (MM "Therapeutics") OR (MM "Patient Comfort") OR (MM "Moving and Lifting Patients") OR (MM "Refusal to Treat") OR (MM "Patient-Centered Care") OR (MM "Comprehensive Health Care") OR (MM "Education, Continuing") OR (MM "Education, Public Health Professional") OR (MM "Preceptorship") OR (MM "Mentoring") OR (MM "Interprofessional Education") OR (MM "Internship, Nonmedical") OR (MM "Education, Pharmacy") OR (MM "Education, Nursing") OR (MM "Education, Medical") OR (MM "Education, Graduate") OR (MM "Education, Medical, Continuing") OR (MM "Education, Pharmacy, Continuing") OR (MM "Education, Nursing, Continuing") OR (MM "Education, Professional") OR (MM "Education, Professional, Retraining") OR (MM "Clinical Clerkship") OR (MM "Education") OR (MM "Teacher Training") OR (MM "Exercise Therapy") OR (MM "Rehabilitation") OR TI ( (Continuity of Patient Care) OR (Patient Care) OR (Patient Transfer) OR (Withholding Treatment) OR (Transitional Care) OR (Time-to-Treatment) OR (Terminal Care) OR (Subacute Care) OR (Preoperative Care) OR (Patient Positioning) OR (Palliative Care) OR (Nursing Care) OR (Night Care) OR (Life Support Care) OR (Institutionalization) OR (Hospitalization) OR (Critical Care) OR (Episode of Care) OR (Duration of Therapy) OR (Delayed Diagnosis) OR (Custodial Care) OR (Retention in Care) OR (Patient Handoff) OR (Aftercare) OR (Therapeutics) OR (Patient Comfort) OR (Moving and Lifting Patients) OR (Refusal to Treat) OR (Patient-Centered Care) OR (Comprehensive Health Care) OR (Education, Continuing) OR (Education, Public Health Professional) OR (Preceptorship) OR (Mentoring) OR (Interprofessional Education) OR (Internship, Nonmedical) OR (Education, Pharmacy) OR (Education, Nursing) OR (Education, Medical) OR (Education, Graduate) OR (Education, Medical, Continuing) OR (Education, Pharmacy, Continuing) OR (Education, Nursing, Continuing) OR (Education, Professional) OR (Education, Professional, Retraining) OR (Clinical Clerkship) OR (Education) OR (Teacher Training) OR (Exercise Therapy) OR (Rehabilitation) ) OR AB ( (Continuity of Patient Care) OR (Patient Care) OR (Patient Transfer) OR (Withholding Treatment) OR (Transitional Care) OR (Time-to-Treatment) OR (Terminal Care) OR (Subacute Care) OR (Preoperative Care) OR (Patient Positioning) OR (Palliative Care) OR (Nursing Care) OR (Night Care) OR (Life Support Care) OR (Institutionalization) OR (Hospitalization) OR (Critical Care) OR (Episode of Care) OR (Duration of Therapy) OR (Delayed Diagnosis) OR (Custodial Care) OR (Retention in Care) OR (Patient Handoff) OR (Aftercare) OR (Therapeutics) OR (Patient Comfort) OR (Moving and Lifting Patients) OR (Refusal to Treat) OR (Patient-Centered Care) OR (Comprehensive Health Care) OR (Education, Continuing) OR (Education, Public Health Professional) OR (Preceptorship) OR (Mentoring) OR (Interprofessional Education) OR (Internship, Nonmedical) OR (Education, Pharmacy) OR (Education, Nursing) OR (Education, Medical) OR (Education, Graduate) OR (Education, Medical, Continuing) OR (Education, Pharmacy, Continuing) OR (Education, Nursing, Continuing) OR (Education, Professional) OR (Education, Professional, Retraining) OR (Clinical Clerkship) OR (Education) OR (Teacher Training) OR (Exercise Therapy) OR (Rehabilitation) )  **OR**  **Adaptability**  Capacité de s’adapter aux attentes, aux valeurs et aux droits des usagers.  (MM "Virus Shedding") OR (MM "Stress, Physiological") OR (MM "Pharmacological and Toxicological Phenomena") OR (MM "Organism Hydration Status") OR (MM "Longevity") OR (MM "Movement") OR (MM "Homeostasis") OR (MM "Electrophysiological Phenomena") OR (MM "Diet, Food, and Nutrition") OR (MM "Body Constitution") OR (MM "Bacterial Shedding") OR (MM "Anatomic Variation") OR (MM "Acclimatization") OR (MM "Adaptation, Physiological") OR (MM "Thermosensing") OR (MM "Body Temperature Regulation") OR (MM "Host Adaptation") OR (MM "Adaptation, Biological") OR (MM "Psychology, Social") OR (MH "Personality") OR (MM "Psychosocial Functioning") OR (MM "Temperance") OR (MM "Neurobehavioral Manifestations") OR (MM "Motivation") OR (MM "Mental Competency") OR (MM "Emotions") OR (MM "Defense Mechanisms") OR (MM "Behavior") OR (MM "Attitude") OR (MM "Human Characteristics") OR (MM "Human Development") OR (MM "Sense of Coherence") OR (MM "Posttraumatic Growth, Psychological") OR (MM "Feedback, Psychological") OR (MM "Emotional Adjustment") OR (MM "Adaptation, Psychological") OR (MM "Behavior and Behavior Mechanisms") OR (MM "General Adaptation Syndrome") OR (MM "Social Behavior Disorders") OR (MM "Sports for Persons with Disabilities") OR (MM "Walking") OR (MM "Recreation") OR (MM "Human Activities") OR (MM "Water Sports") OR (MM "Leisure Activities") OR TI ( (Virus Shedding) OR (Stress, Physiological) OR (Pharmacological and Toxicological Phenomena) OR (Organism Hydration Status) OR (Longevity) OR (Movement) OR (Homeostasis) OR (Electrophysiological Phenomena) OR (Diet, Food, and Nutrition) OR (Body Constitution) OR (Bacterial Shedding) OR (Anatomic Variation) OR (Acclimatization) OR (Adaptation, Physiological) OR (Thermosensing) OR (Body Temperature Regulation) OR (Host Adaptation) OR (Adaptation, Biological) OR (Psychology, Social) OR (Personality) OR (Psychosocial Functioning) OR (Temperance) OR (Neurobehavioral Manifestations) OR (Motivation) OR (Mental Competency) OR (Emotions) OR (Defense Mechanisms) OR (Behavior) OR (Attitude) OR (Human Characteristics) OR (Human Development) OR (Sense of Coherence) OR (Posttraumatic Growth, Psychological) OR (Feedback, Psychological) OR (Emotional Adjustment) OR (Adaptation, Psychological) OR (Behavior and Behavior Mechanisms) OR (General Adaptation Syndrome) OR (Social Behavior Disorders) OR (Sports for Persons with Disabilities) OR (Walking) OR (Recreation) OR (Human Activities) OR (Water Sports) OR (Leisure Activities) ) OR AB ( (Virus Shedding) OR (Stress, Physiological) OR (Pharmacological and Toxicological Phenomena) OR (Organism Hydration Status) OR (Longevity) OR (Movement) OR (Homeostasis) OR (Electrophysiological Phenomena) OR (Diet, Food, and Nutrition) OR (Body Constitution) OR (Bacterial Shedding) OR (Anatomic Variation) OR (Acclimatization) OR (Adaptation, Physiological) OR (Thermosensing) OR (Body Temperature Regulation) OR (Host Adaptation) OR (Adaptation, Biological) OR (Psychology, Social) OR (Personality) OR (Psychosocial Functioning) OR (Temperance) OR (Neurobehavioral Manifestations) OR (Motivation) OR (Mental Competency) OR (Emotions) OR (Defense Mechanisms) OR (Behavior) OR (Attitude) OR (Human Characteristics) OR (Human Development) OR (Sense of Coherence) OR (Posttraumatic Growth, Psychological) OR (Feedback, Psychological) OR (Emotional Adjustment) OR (Adaptation, Psychological) OR (Behavior and Behavior Mechanisms) OR (General Adaptation Syndrome) OR (Social Behavior Disorders) OR (Sports for Persons with Disabilities) OR (Walking) OR (Recreation) OR (Human Activities) OR (Water Sports) OR (Leisure Activities) )  **Satisfaction**  (MM "Job Satisfaction") OR (MM "Vocational Guidance") OR (MM "Psychological Phenomena") OR (MM "Personal Satisfaction") OR (MM "Spatial Behavior") OR (MM "Risk-Taking") OR (MM "Multitasking Behavior") OR (MM "Motor Activity") OR (MM "Inhibition, Psychological") OR (MM "Information Seeking Behavior") OR (MM "Health Behavior") OR (MM "Harm Reduction") OR (MM "Habits") OR (MM "Illness Behavior") OR (MM "Communication") OR (MM "Human-Animal Interaction") OR (MM "Feeding Behavior") OR (MM "Escape Reaction") OR (MM "Exploratory Behavior") OR (MM "Dangerous Behavior") OR (MM "Behavioral Symptoms") OR (MM "Accident Proneness") OR (MM "Treatment Adherence and Compliance") OR (MM "Patient Satisfaction") OR (MM "Treatment Refusal") OR (MM "Patient Preference") OR (MM "Patient Acceptance of Health Care") OR (MM "Attitude to Health") OR (MM "Public Relations") OR (MM "Interinstitutional Relations") OR (MM "Interdepartmental Relations") OR (MM "Community-Institutional Relations") OR (MM "Hospital-Physician Relations") OR (MM "Hospital-Patient Relations") OR (MM "Treatment Switching") OR (MM "Technology Assessment, Biomedical") OR (MM "Program Evaluation") OR (MM "Outcome and Process Assessment, Health Care") OR (MM "Root Cause Analysis") OR TI ( (Job Satisfaction) OR (Vocational Guidance) OR (Psychological Phenomena) OR (Personal Satisfaction) OR (Spatial Behavior) OR (Risk-Taking) OR (Multitasking Behavior) OR (Motor Activity) OR (Inhibition, Psychological) OR (Information Seeking Behavior) OR (Health Behavior) OR (Harm Reduction) OR (Habits) OR (Illness Behavior) OR (Communication) OR (Human-Animal Interaction) OR (Feeding Behavior) OR (Escape Reaction) OR (Exploratory Behavior) OR (Dangerous Behavior) OR (Behavioral Symptoms) OR (Accident Proneness) OR (Treatment Adherence and Compliance) OR (Patient Satisfaction) OR (Treatment Refusal) OR (Patient Preference) OR (Patient Acceptance of Health Care) OR (Attitude to Health) OR (Public Relations) OR (Interinstitutional Relations) OR (Interdepartmental Relations) OR (Community-Institutional Relations) OR (Hospital-Physician Relations) OR (Hospital-Patient Relations) OR (Treatment Switching) OR (Technology Assessment, Biomedical) OR (Program Evaluation) OR (Outcome and Process Assessment, Health Care) OR (Root Cause Analysis) ) OR AB ( (Job Satisfaction) OR (Vocational Guidance) OR (Psychological Phenomena) OR (Personal Satisfaction) OR (Spatial Behavior) OR (Risk-Taking) OR (Multitasking Behavior) OR (Motor Activity) OR (Inhibition, Psychological) OR (Information Seeking Behavior) OR (Health Behavior) OR (Harm Reduction) OR (Habits) OR (Illness Behavior) OR (Communication) OR (Human-Animal Interaction) OR (Feeding Behavior) OR (Escape Reaction) OR (Exploratory Behavior) OR (Dangerous Behavior) OR (Behavioral Symptoms) OR (Accident Proneness) OR (Treatment Adherence and Compliance) OR (Patient Satisfaction) OR (Treatment Refusal) OR (Patient Preference) OR (Patient Acceptance of Health Care) OR (Attitude to Health) OR (Public Relations) OR (Interinstitutional Relations) OR (Interdepartmental Relations) OR (Community-Institutional Relations) OR (Hospital-Physician Relations) OR (Hospital-Patient Relations) OR (Treatment Switching) OR (Technology Assessment, Biomedical) OR (Program Evaluation) OR (Outcome and Process Assessment, Health Care) OR (Root Cause Analysis) )  OR  ***Viability***  Capacité, considérée sous l’angle des ressources humaines, matérielles, financières, technologiques et informationnelles, de répondre aux besoins présents et futurs de la population.  ***Resource mobilization***  (MM "Social Mobility") OR (MM "Career Mobility") OR (MM "Workplace") OR (MM "Personnel Downsizing") OR (MH "Employment, Supported") OR (MM "Return to Work") OR (MM "Employment") OR (MM "Socioeconomic Factors") (MM "Occupations") OR (MH "Health Care Facilities, Manpower, and Services") OR (MM "Telephone") OR (MM "Text Messaging") OR (MM "Smartphone") OR (MM "Cell Phone") OR (MM "Answering Services") OR (MM "Telecommunications") OR (MM "Communications Media") OR (MM "Personnel Delegation") OR (MM "Employee Incentive Plans") OR (MM "Employee Grievances") OR TI ( (Social Mobility) OR (Career Mobility) OR (Workplace) OR (Personnel Downsizing) OR (Employment, Supported) OR (Return to Work) OR (Employment) OR (Socioeconomic Factors) OR (Occupations) OR (Health Care Facilities, Manpower, and Services) OR (Telephone) OR (Text Messaging) OR (Smartphone) OR (Cell Phone) OR (Answering Services) OR (Telecommunications) OR (Communications Media) OR (Personnel Delegation)) OR (Employee Incentive Plans) OR (Employee Grievances)) AB ( (Social Mobility) OR (Career Mobility) OR (Workplace) OR (Personnel Downsizing) OR (Employment, Supported) OR (Return to Work) OR (Employment) OR (Socioeconomic Factors) OR (Occupations) OR (Health Care Facilities, Manpower, and Services) OR (Telephone) OR (Text Messaging) OR (Smartphone) OR (Cell Phone) OR (Answering Services) OR (Telecommunications) OR (Communications Media) OR (Personnel Delegation) OR (Employee Incentive Plans) OR (Employee Grievances))  **OR**  ***Resource management***  (MM "Practice Management") OR (MM "Professional Practice") OR (MM "Professional Staff Committees") OR (MM "Practice Management, Medical") OR (MM "Management Service Organizations") OR (MM "Professional Corporations") OR (MM "Partnership Practice") OR (MM "Organization and Administration") OR (MM "Institutional Practice") OR (MM "Group Practice") OR (MM "Health Maintenance Organizations") OR (MM "Independent Practice Associations") OR (MM "Organizational Innovation") OR (MM "Change Management") OR (MM "Entrepreneurship") OR (MM "Recycling") OR (MM "Time Management") OR (MM "Task Performance and Analysis") OR (MM "Psychology, Applied") OR (MM "Presenteeism") OR (MM "Absenteeism") OR (MM "Risk Assessment") OR (MM "Risk Evaluation and Mitigation") OR (MM "Risk Sharing, Financial") OR (MM "Shared Governance, Nursing") OR (MM "Total Quality Management") OR (MM "Planning Techniques") OR (MM "Pharmacy Administration") OR (MM "Personnel Management") OR (MM "Organizational Objectives") OR (MM "Organizational Culture") OR (MM "Organizational Affiliation") OR (MM "Models, Organizational") OR (MM "Workforce") OR (MM "Decision Making, Organizational") OR (MM "Clinical Governance") OR (MM "Capacity Building") OR (MM "Management Information Systems") OR (MM "Benchmarking") OR (MM "Management Audit") OR (MM "Knowledge Management") OR (MM "Institutional Management Teams") OR (MM "Health Facility Administration") OR (MM "Governing Board") OR (MM "Annual Reports as Topic") OR (MM "Nurse Administrators") OR (MM "Health Facility Administrators") OR (MM "Case Managers") OR (MM "Administrative Personnel") OR (MM "Occupational Groups") OR (MM "Psychotherapists") OR (MM "Physicians") OR (MM "Pharmacists") OR (MM "Occupational Therapists") OR (MM "Nursing Staff") OR (MM "Medical Staff") OR (MM "Infection Control Practitioners") OR (MM "Health Educators") OR (MM "Dentists") OR (MM "Audiologists") OR (MM "Caregivers") OR (MM "Epidemiologists") OR (MM "Nutritionists") OR (MM "Optometrists") OR (MM "Physical Therapists") OR (MM "Self-Management") OR (MM "Forms and Records Control") OR (MM "Office Management") OR (MM "Management Quality Circles") OR (MM "Personnel Turnover") OR (MM "Personnel Staffing and Scheduling") OR (MM "Personnel Loyalty") OR (MM "Personnel Selection") OR (MM "Job Description") OR (MM "Job Application") OR (MM "Employee Discipline") OR TI ( (Practice Management) OR (Professional Practice) OR (Professional Staff Committees) OR (Practice Management, Medical) OR (Management Service Organizations) OR (Professional Corporations) OR (Partnership Practice) OR (Organization and Administration) OR (Institutional Practice) OR (Group Practice) OR (Health Maintenance Organizations) OR (Independent Practice Associations) OR Organizational Innovation) OR (Change Management) OR (Entrepreneurship) OR (Recycling) OR (Time Management) OR (Task Performance and Analysis) OR (Psychology, Applied) OR (Presenteeism) OR (Absenteeism) OR (Risk Assessment) OR (Risk Evaluation and Mitigation) OR (Risk Sharing, Financial) OR (Shared Governance, Nursing) OR (Total Quality Management) OR (Planning Techniques) OR (Pharmacy Administration) OR (Personnel Management) OR (Organizational Objectives) OR (Organizational Culture) OR (Organizational Affiliation) OR (Models, Organizational) OR (Workforce) OR (Decision Making, Organizational) OR (Clinical Governance) OR (Capacity Building) OR (Management Information Systems) OR (Benchmarking) OR (Management Audit) OR (Knowledge Management) OR (Institutional Management Teams) OR (Health Facility Administration) OR (Governing Board) OR (Annual Reports as Topic) OR (Nurse Administrators) OR (Health Facility Administrators) OR (Case Managers) OR (Administrative Personnel) OR (Occupational Groups) OR (Psychotherapists) OR (Physicians) OR (Pharmacists) OR (Occupational Therapists) OR (Nursing Staff) OR (Medical Staff) OR (Infection Control Practitioners) OR (Health Educators) OR (Dentists) OR (Audiologists) OR (Caregivers) OR (Epidemiologists) OR (Nutritionists) OR (Optometrists) OR (Physical Therapists) OR (Self-Management) OR (Forms and Records Control) OR (Office Management) OR (Management Quality Circles) OR (Personnel Turnover) OR (Personnel Staffing and Scheduling) OR (Personnel Loyalty) OR (Personnel Selection) OR (Job Description) OR (Job Application) OR (Employee Discipline)) OR AB ( (Practice Management) OR (Professional Practice) OR (Professional Staff Committees) OR (Practice Management, Medical) OR (Management Service Organizations) OR (Professional Corporations) OR (Partnership Practice) OR (Organization and Administration) OR (Institutional Practice) OR (Group Practice) OR (Health Maintenance Organizations) OR (Independent Practice Associations) OR Organizational Innovation) OR (Change Management) OR (Entrepreneurship) OR (Recycling) OR (Time Management) OR (Task Performance and Analysis) OR (Psychology, Applied) OR (Presenteeism) OR (Absenteeism) OR (Risk Assessment) OR (Risk Evaluation and Mitigation) OR (Risk Sharing, Financial) OR (Shared Governance, Nursing) OR (Total Quality Management) OR (Planning Techniques) OR (Pharmacy Administration) OR (Personnel Management) OR (Organizational Objectives) OR (Organizational Culture) OR (Organizational Affiliation) OR (Models, Organizational) OR (Workforce) OR (Decision Making, Organizational) OR (Clinical Governance) OR (Capacity Building) OR (Management Information Systems) OR (Benchmarking) OR (Management Audit) OR (Knowledge Management) OR (Institutional Management Teams) OR (Health Facility Administration) OR (Governing Board) OR (Annual Reports as Topic) OR (Nurse Administrators) OR (Health Facility Administrators) OR (Case Managers) OR (Administrative Personnel) OR (Occupational Groups) OR (Psychotherapists) OR (Physicians) OR (Pharmacists) OR (Occupational Therapists) OR (Nursing Staff) OR (Medical Staff) OR (Infection Control Practitioners) OR (Health Educators) OR (Dentists) OR (Audiologists) OR (Caregivers) OR (Epidemiologists) OR (Nutritionists) OR (Optometrists) OR (Physical Therapists) OR (Self-Management) OR (Forms and Records Control) OR (Office Management) OR (Management Quality Circles) OR (Personnel Turnover) OR (Personnel Staffing and Scheduling) OR (Personnel Loyalty) OR (Personnel Selection) OR (Job Description) OR (Job Application) OR (Employee Discipline))  **OR**  **Structures (remplacé par organizational structures)**  "(MM "Organizational Objectives") OR (MM "Organization and Administration") OR (MM "Shared Governance, Nursing") OR (MM "Time Management") OR (MM "Voluntary Programs") OR (MM "Total Quality Management") OR (MM "Security Measures") OR (MM "Risk Management") OR (MM "Records") OR (MM "Public Relations") OR (MM "Public Health Administration") OR (MM "Program Development") OR (MM "Professional Practice") OR (MM "Planning Techniques") OR (MM "Pharmacy Administration") OR (MM "Personnel Management") OR (MM "Patient Identification Systems") OR (MM "Ownership") OR (MM "Organizational Innovation") OR (MM "Organizational Culture") OR (MM "Organizational Affiliation") OR (MM "Multi-Institutional Systems") OR (MM "Models, Organizational") OR (MM "Medication Systems") OR (MM "Workforce") OR (MM "Veterans Health Services") OR (MM "Military Health Services") OR (MM "Mandatory Programs") OR (MM "Decision Making, Organizational") OR (MM "Clinical Governance") OR (MM "Capacity Building") OR (MM "Constitution and Bylaws") OR (MM "Committee Membership") OR (MM "Management Information Systems") OR (MM "Management Audit") OR (MM "Knowledge Management") OR (MM "Institutional Management Teams") OR (MM "Health Facility Administration") OR (MM "Governing Board") OR (MM "Fee Schedules") OR (MM "Facilities and Services Utilization") OR (MM "Eligibility Determination") OR (MM "Efficiency") OR (MM "Appointments and Schedules") OR (MM "Annual Reports as Topic") OR TI ((Organizational Objectives) OR (Organization and Administration) OR (Shared Governance, Nursing) OR (Time Management) OR (Voluntary Programs) OR (Total Quality Management) OR (Security Measures) OR (Risk Management) OR (Records) OR (Public Relations) OR (Public Health Administration) OR (Program Development) OR (Professional Practice) OR (Planning Techniques) OR (Pharmacy Administration) OR (Personnel Management) OR (Patient Identification Systems) OR (Ownership) OR (Organizational Innovation) OR (Organizational Culture) OR (Organizational Affiliation) OR (Multi-Institutional Systems) OR (Models, Organizational) OR (Medication Systems) OR (Workforce) OR (Veterans Health Services) OR (Military Health Services) OR (Mandatory Programs) OR (Decision Making, Organizational) OR (Clinical Governance) OR (Capacity Building) OR (Constitution and Bylaws) OR (Committee Membership) OR (Management Information Systems) OR (Management Audit) OR (Knowledge Management) OR (Institutional Management Teams) OR (Health Facility Administration) OR (Governing Board) OR (Fee Schedules) OR (Facilities and Services Utilization) OR (Eligibility Determination) OR (Efficiency) OR (Appointments and Schedules) OR (Annual Reports as Topic)) OR AB ((Organizational Objectives) OR (Organization and Administration) OR (Shared Governance, Nursing) OR (Time Management) OR (Voluntary Programs) OR (Total Quality Management) OR (Security Measures) OR (Risk Management) OR (Records) OR (Public Relations) OR (Public Health Administration) OR (Program Development) OR (Professional Practice) OR (Planning Techniques) OR (Pharmacy Administration) OR (Personnel Management) OR (Patient Identification Systems) OR (Ownership) OR (Organizational Innovation) OR (Organizational Culture) OR (Organizational Affiliation) OR (Multi-Institutional Systems) OR (Models, Organizational) OR (Medication Systems) OR (Workforce) OR (Veterans Health Services) OR (Military Health Services) OR (Mandatory Programs) OR (Decision Making, Organizational) OR (Clinical Governance) OR (Capacity Building) OR (Constitution and Bylaws) OR (Committee Membership) OR (Management Information Systems) OR (Management Audit) OR (Knowledge Management) OR (Institutional Management Teams) OR (Health Facility Administration) OR (Governing Board) OR (Fee Schedules) OR (Facilities and Services Utilization) OR (Eligibility Determination) OR (Efficiency) OR (Appointments and Schedules) OR (Annual Reports as Topic))  **Results (outcomes)**  **Care procedures**  "(MM "Endovascular Procedures") OR (MM "Catheterization, Peripheral") OR (MM "Catheterization, Central Venous") OR (MM "Percutaneous Coronary Intervention") OR (MM "Contraindications") OR (MM "Contraindications, Procedure") OR (MM "Contraindications, Drug") OR (MM "Therapeutics") OR (MM "Unnecessary Procedures") OR (MM "Health Services Misuse") OR (MM "Medical Overuse") OR (MM "Health Services") OR (MM "Health Care Facilities, Manpower, and Services") OR (MM "Attitude to Health") OR (MM "Delivery of Health Care") OR (MM "Health Care Quality, Access, and Evaluation") OR (MM "Self Care") OR (MM "Respiratory Therapy") OR (MM "Rehabilitation") OR (MM "Prosthesis Fitting") OR (MM "Physical Therapy Modalities") OR (MM "Patient Isolation") OR (MM "Patient Care Bundles") OR (MM "Patient Care") OR (MM "Pain Management") OR (MM "Orthoptics") OR (MM "Obesity Management") OR (MM "Nutrition Therapy") OR (MM "Hygiene") OR (MM "Hemostatic Techniques") OR (MM "Feeding Methods") OR (MM "Emergency Treatment") OR (MM "Drug Therapy") OR (MM "Drainage") OR (MM "Decompression") OR (MM "Conservative Treatment") OR (MM "Complementary Therapies") OR (MM "Bed Rest") OR (MM "Airway Management") OR (MM "Acoustic Stimulation") OR (MM "Secondary Prevention") OR (MM "Glycemic Control") OR (MM "Catheterization") OR (MM "Behavior Control") OR (MM "Orthokeratologic Procedures") OR (MM "Pain, Procedural") OR (MM "Pain") OR (MM "Headache") OR (MM "Pelvic Pain") OR (MM "Nociceptive Pain") OR (MM "Neuralgia") OR (MM "Neck Pain") OR (MM "Musculoskeletal Pain") OR (MM "Metatarsalgia") OR (MM "Facial Pain") OR (MM "Cancer Pain") OR (MM "Pain, Referred") OR (MM "Pain, Postoperative") OR (MM "Chest Pain") OR (MM "Arthralgia") OR (MM "Back Pain") OR (MM "Acute Pain") OR (MM "Abdominal Pain") OR (MM "Chronic Pain") OR (MM "Neurologic Manifestations") OR (MM "Signs and Symptoms") OR (MM "Renal Colic") OR (MM "Subacute Care") OR (MM "Transitional Care") OR (MM "Terminal Care") OR (MM "Shared Medical Appointments") OR (MM "Patient Positioning") OR (MM "Palliative Care") OR (MM "Nursing Care") OR (MM "Night Care") OR (MM "Long-Term Care") OR (MM "Life Support Care") OR (MM "Institutionalization") OR (MM "Hospitalization") OR (MM "Episode of Care") OR (MM "Continuity of Patient Care") OR (MM "Ambulatory Care") OR (MM "Withholding Treatment") OR (MM "Duration of Therapy") OR (MM "Delayed Diagnosis") OR (MM "Critical Care") OR (MM "Time-to-Treatment") OR (MM "Moving and Lifting Patients") OR (MM "Patient Comfort") OR (MM "Day Care, Medical") OR (MH "Preoperative Care") OR (MM "Home Nursing") OR (MM "Respite Care") OR (MM "Community Health Services") OR (MH "Postoperative Care") OR (MM "Preoperative Exercise") OR (MH "Orthopedic Procedures") OR "care procedures" OR TI ((Endovascular Procedures) OR (Catheterization, Peripheral) OR (Catheterization, Central Venous) OR (Percutaneous Coronary Intervention) OR (Contraindications) OR (Contraindications, Procedure) OR (Contraindications, Drug) OR (Therapeutics) OR (Unnecessary Procedures) OR (Health Services Misuse) OR (Medical Overuse) OR (Health Services) OR (Health Care Facilities, Manpower, and Services) OR (Attitude to Health) OR (Delivery of Health Care) OR (Health Care Quality, Access, and Evaluation) OR (Self Care) OR (Respiratory Therapy) OR (Rehabilitation) OR (Prosthesis Fitting) OR (Physical Therapy Modalities) OR (Patient Isolation) OR (Patient Care Bundles) OR (Patient Care) OR (Pain Management) OR (Orthoptics) OR (Obesity Management) OR (Nutrition Therapy) OR (Hygiene) OR (Hemostatic Techniques) OR (Feeding Methods) OR (Emergency Treatment) OR (Drug Therapy) OR (Drainage) OR (Decompression) OR (Conservative Treatment) OR (Complementary Therapies) OR (Bed Rest) OR (Airway Management) OR (Acoustic Stimulation) OR (Secondary Prevention) OR (Glycemic Control) OR (Catheterization) OR (Behavior Control) OR (Orthokeratologic Procedures) OR (Pain, Procedural) OR (Pain) OR (Headache) OR (Pelvic Pain) OR (Nociceptive Pain) OR (Neuralgia) OR (Neck Pain) OR (Musculoskeletal Pain) OR (Metatarsalgia) OR (Facial Pain) OR (Cancer Pain) OR (Pain, Referred) OR (Pain, Postoperative) OR (Chest Pain) OR (Arthralgia) OR (Back Pain) OR (Acute Pain) OR (Abdominal Pain) OR (Chronic Pain) OR (Neurologic Manifestations) OR (Signs and Symptoms) OR (Renal Colic) OR (Subacute Care) OR (Transitional Care) OR (Terminal Care) OR (Shared Medical Appointments) OR (Patient Positioning) OR (Palliative Care) OR (Nursing Care) OR (Night Care) OR (Long-Term Care) OR (Life Support Care) OR (Institutionalization) OR (Hospitalization) OR (Episode of Care) OR (Continuity of Patient Care) OR (Ambulatory Care) OR (Withholding Treatment) OR (Duration of Therapy) OR (Delayed Diagnosis) OR (Critical Care) OR (Time-to-Treatment) OR (Moving and Lifting Patients) OR (Patient Comfort) OR (Day Care, Medical) OR (Preoperative Care) OR (Home Nursing) OR (Respite Care) OR (Community Health Services) OR (Postoperative Care) OR (Preoperative Exercise) OR (Orthopedic Procedures) OR (care procedures)) OR AB ((Endovascular Procedures) OR (Catheterization, Peripheral) OR (Catheterization, Central Venous) OR (Percutaneous Coronary Intervention) OR (Contraindications) OR (Contraindications, Procedure) OR (Contraindications, Drug) OR (Therapeutics) OR (Unnecessary Procedures) OR (Health Services Misuse) OR (Medical Overuse) OR (Health Services) OR (Health Care Facilities, Manpower, and Services) OR (Attitude to Health) OR (Delivery of Health Care) OR (Health Care Quality, Access, and Evaluation) OR (Self Care) OR (Respiratory Therapy) OR (Rehabilitation) OR (Prosthesis Fitting) OR (Physical Therapy Modalities) OR (Patient Isolation) OR (Patient Care Bundles) OR (Patient Care) OR (Pain Management) OR (Orthoptics) OR (Obesity Management) OR (Nutrition Therapy) OR (Hygiene) OR (Hemostatic Techniques) OR (Feeding Methods) OR (Emergency Treatment) OR (Drug Therapy) OR (Drainage) OR (Decompression) OR (Conservative Treatment) OR (Complementary Therapies) OR (Bed Rest) OR (Airway Management) OR (Acoustic Stimulation) OR (Secondary Prevention) OR (Glycemic Control) OR (Catheterization) OR (Behavior Control) OR (Orthokeratologic Procedures) OR (Pain, Procedural) OR (Pain) OR (Headache) OR (Pelvic Pain) OR (Nociceptive Pain) OR (Neuralgia) OR (Neck Pain) OR (Musculoskeletal Pain) OR (Metatarsalgia) OR (Facial Pain) OR (Cancer Pain) OR (Pain, Referred) OR (Pain, Postoperative) OR (Chest Pain) OR (Arthralgia) OR (Back Pain) OR (Acute Pain) OR (Abdominal Pain) OR (Chronic Pain) OR (Neurologic Manifestations) OR (Signs and Symptoms) OR (Renal Colic) OR (Subacute Care) OR (Transitional Care) OR (Terminal Care) OR (Shared Medical Appointments) OR (Patient Positioning) OR (Palliative Care) OR (Nursing Care) OR (Night Care) OR (Long-Term Care) OR (Life Support Care) OR (Institutionalization) OR (Hospitalization) OR (Episode of Care) OR (Continuity of Patient Care) OR (Ambulatory Care) OR (Withholding Treatment) OR (Duration of Therapy) OR (Delayed Diagnosis) OR (Critical Care) OR (Time-to-Treatment) OR (Moving and Lifting Patients) OR (Patient Comfort) OR (Day Care, Medical) OR (Preoperative Care) OR (Home Nursing) OR (Respite Care) OR (Community Health Services) OR (Postoperative Care) OR (Preoperative Exercise) OR (Orthopedic Procedures) OR (care procedures)) |

**AND**

| *Long term care*  (MM "Long Term Care") OR "long term care" OR (MM "Nursing Home Patients") OR (MM "Hospice Patients") OR TI ( ‘Long-Term Care' or 'Assisted-Living Facilities' or 'long-term-care facility' or 'Homes for the Aged' or 'Nursing Homes' or 'nursing home' or 'long-term care' or 'retirement home' or ‘hospice patients’ or ‘hospice care’) OR AB ( 'Long-Term Care' or 'Assisted-Living Facilities' or 'long-term-care facility' or 'Homes for the Aged' or 'Nursing Homes' or 'nursing home' or 'long-term care' or 'retirement home' or ‘hospice patients’ or ‘hospice care’) |
| --- |

**AND**

| *COVID-19*  TI (covid-19 or coronavirus or 2019-ncov or sars-cov-2 or cov-19 or covid) OR AB (covid-19 or coronavirus or 2019-ncov or sars-cov-2 or cov-19 or covid) |
| --- |
