# Supplementary material for: Identifying performance factors of long-term care facilities in the context of the COVID-19 pandemic: a scoping review protocol
Source: Syst Rev. 2022 Sep 23;11:203. doi: 10.1186/s13643-022-02069-1 (PMC9502645; doi:10.1186/s13643-022-02069-1)
Supplement: Supplementary file 5 — Additional file 5: CAIRN. [file 13643_2022_2069_MOESM5_ESM.docx]

**Supplementary File 5**

**CAIRN Search Strategy**

TI (covid-19 OU coronavirus OU 2019-ncov OU sars-cov-2 OU cov-19) ET TI (EHPAD OU établissements hospitaliers pour personnes âgées dépendantes OU foyers de soins) ET tout (résultats OU procédures de soins OU structure organisationnelle OU gestion des ressources OU continuité OU efficacité OU efficience OU sécurité OU accessibilité OU équité OU adaptabilité OU satisfaction OU mobilisation des ressources)

**2 résultats**

TOUT (covid-19 OU coronavirus OU 2019-ncov OU sars-cov-2 OU cov-19) ET TOUT (EHPAD OU établissements hospitaliers pour personnes âgées dépendantes OU foyers de soins) ET TOUT (résultats OU procédures de soins OU structure organisationnelle OU gestion des ressources OU continuité OU efficacité OU efficience OU sécurité OU accessibilité OU équité OU adaptabilité OU satisfaction OU mobilisation des ressources)

**215 résultats**

((covid-19) OU (coronavirus) OU (2019-ncov) OU (sars-cov-2) OU (cov-19))

((EHPAD) OU (établissements hospitaliers pour personnes âgées dépendantes) OU (foyers de soins))

((résultats) OU (procédures de soins) OU (structure organisationnelle) OU (gestion des ressources) OU (continuité) OU (efficacité) OU (efficience) OU (sécurité) OU (accessibilité) OU (équité) OU (adaptabilité) OU (satisfaction) OU (mobilisation des ressources))
