# Supplementary material for: Identifying performance factors of long-term care facilities in the context of the COVID-19 pandemic: a scoping review protocol
Source: Syst Rev. 2022 Sep 23;11:203. doi: 10.1186/s13643-022-02069-1 (PMC9502645; doi:10.1186/s13643-022-02069-1)
Supplement: Supplementary file 6 — Additional file 6: Science Direct. [file 13643_2022_2069_MOESM6_ESM.docx]

**Supplementary File 6**

**Science Direct Search Strategy**

ALL ((outcomes) OR (care procedures) OR (organizational structure) OR (resource management) OR (continuity) OR (efficiency) OR (security) OR (accessibility) OR (equity)) Title, abstract, keywords: ((Long-Term Care) OR (Assisted-Living Facilities) OR (long-term-care facility) OR (Homes for the Aged) OR (Nursing Homes) OR (nursing home) OR (long-term care) OR (retirement home)) AND (COVID-19)

**739 results**

ALL ((efficacy) OR (effectiveness) OR (safety) OR (adaptability) OR (satisfaction) OR (resource mobilization)) Title, abstract, keywords: ((Long-Term Care) OR (Assisted-Living Facilities) OR (long-term-care facility) OR (Homes for the Aged) OR (Nursing Homes) OR (nursing home) OR (long-term care) OR (retirement home)) AND (COVID-19)

**477 results**

**Total: 1,216 results**
