# Supplementary material for: Identifying performance factors of long-term care facilities in the context of the COVID-19 pandemic: a scoping review protocol
Source: Syst Rev. 2022 Sep 23;11:203. doi: 10.1186/s13643-022-02069-1 (PMC9502645; doi:10.1186/s13643-022-02069-1)
Supplement: Supplementary file 7 — Additional file 7: Web of Science. [file 13643_2022_2069_MOESM7_ESM.docx]

**Supplementary File 7**

**Web of Science Search Strategy**


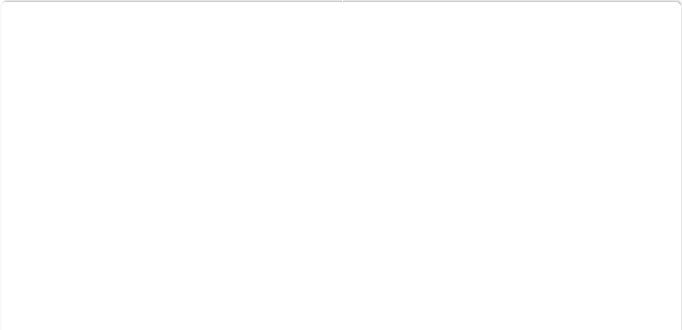

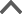

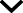

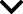

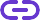

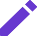

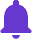

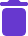

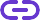

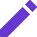

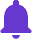

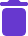

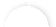

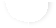


**Type**

**Search Query and Results**

**Database**

**Results**

**Actions**

Search

**#13 AND #10 AND #4**

Web of Science Core Collection

Show editions

[2,308](https://www.webofscience.com/wos/woscc/summary/bc40a27e-e434-489c-b071-a4171465670d-0dbad8e8/relevance/1)

2:05 PM

Search

**#5 OR #6 OR #12**

Web of Science Core Collection

Show editions

[184,284](https://www.webofscience.com/wos/woscc/summary/eb7b4c2c-4807-4e1d-b744-2246aad885e5-0dbad5ec/relevance/1)

2:04 PM

26

**Current session**

Search

Search

Search

Search

Search

2:04 PM | Timespan: 2020-01-01 to 2021-12-31 (Index Date)

2:00 PM

2:00 PM

1:59 PM


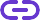

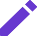
1:59 PM | Timespan: 2020-01-01 to 2021-12-31 (Index Date)

Web of Science Core Collection

Show editions

**ALL=(((Long-Term Care) or (Assisted-Living Facilities) or (long-term-care facility) or (Homes for the Aged) or (Nursing Homes) or (nursing home) or (long-term care) or (retirement home) or (hospice patients) or (hospice care)))**

Web of Science Core Collection


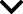


**#4 AND #7 AND #10**

Show editions

Web of Science Core Collection


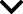


**#8 OR #9**

Show editions

Web of Science Core Collection

**AB=(((covid-19) or (coronavirus) or (2019-ncov) or (sars- cov-2) or (cov-19) or (covid)))**

Show editions

Web of Science Core Collection

**TI=(((covid-19) or (coronavirus) or (2019-ncov) or (sars- cov-2) or (cov-19) or (covid)) )**

Show editions

# [49,236](https://www.webofscience.com/wos/woscc/summary/19cf7199-136c-4493-ae9d-fbb537924054-0dbad434/relevance/1)
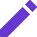


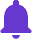

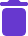


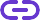

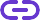
[1,828](https://www.webofscience.com/wos/woscc/summary/28bcab2f-4146-483b-b61d-6414f2dc1466-0dbac71f/relevance/1)
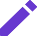


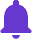

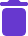


#
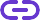
[204,830](https://www.webofscience.com/wos/woscc/summary/0f7ea6a2-37f6-4d77-aaf1-be8104a7d3f2-0dbac415/relevance/1)
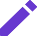


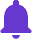

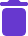


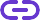
[144,075](https://www.webofscience.com/wos/woscc/summary/f96f5d8d-6627-49f8-8d95-58be46ac45e2-0dbac2ab/relevance/1)
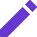


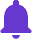

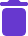


#
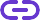
[172,173](https://www.webofscience.com/wos/woscc/summary/1f8f636e-fa85-4a76-af10-d99280bb0f20-0dbac0b2/relevance/1)
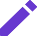


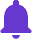

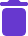


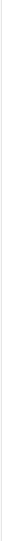


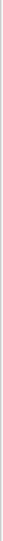

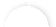

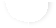


Search

Web of Science Core Collection


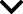


**#5 OR #6**

# [165,877](https://www.webofscience.com/wos/woscc/summary/60ad1101-77b1-44fa-9b19-3e39ceae4cf8-0dbaba13/relevance/1) 26


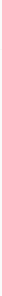
Search

1:57 PM Show editions

Web of Science Core Collection

**AB=(((Long-Term Care) or (Assisted-Living Facilities) or (long-term-care facility) or (Homes for the Aged) or (Nursing Homes) or (nursing home) or (long-term care) or (retirement home) or (hospice patients) or (hospice care)))**

Show editions

1:56 PM


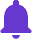

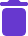


#
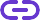
[164,591](https://www.webofscience.com/wos/woscc/summary/3172e208-80fe-4baa-b4b1-06efa29db005-0dbab87f/relevance/1)
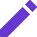


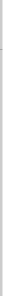

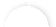

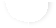


26


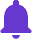

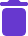


Search


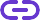
1:56 PM | Timespan: 2020-01-01 to 2021-12-31 (Index Date)

Web of Science Core Collection

Show editions

**TI=(((Long-Term Care) or (Assisted-Living Facilities) or (long-term-care facility) or (Homes for the Aged) or (Nursing Homes) or (nursing home) or (long-term care) or (retirement home) or (hospice patients) or (hospice care)))**

# [4,647](https://www.webofscience.com/wos/woscc/summary/757ebcf3-7acc-492e-b1af-703a9dfa768c-0dbab68d/relevance/1)
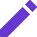


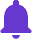

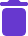


Search


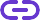

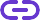
1:53 PM

Web of Science Core Collection

Show editions


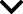


**#1 OR #2 OR #3**

# [8,678,693](https://www.webofscience.com/wos/woscc/summary/e992ce93-3c46-4226-a6e2-fdddbacc462c-0dbaaa5d/relevance/1)
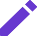


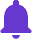

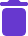


Search

1:44 PM

Web of Science Core Collection

Show editions

**TI=(((outcomes) OR (care procedures) OR (organizational structure) OR (resource management) OR (continuity) OR (eﬀiciency) OR (eﬀicacy) OR (eﬀectiveness) OR (safety) OR (security) OR (accessibility) OR (equity) OR (adaptability) OR (satisfaction) OR (resource mobilization)))**

# [1,696,766](https://www.webofscience.com/wos/woscc/summary/a7533e62-3d21-430c-8402-87ebf2b21aea-0dba87c0/relevance/1)
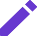


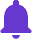

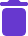


Search


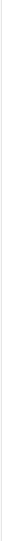

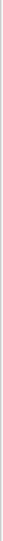

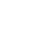

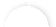

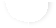


1:42 PM

Web of Science Core Collection

Show editions

**AB=(((outcomes) OR (care procedures) OR (organizational structure) OR (resource management) OR (continuity) OR (eﬀiciency) OR (eﬀicacy) OR (eﬀectiveness) OR (safety) OR (security) OR (accessibility) OR (equity) OR (adaptability) OR (satisfaction) OR (resource mobilization)))**

# [6,468,879](https://www.webofscience.com/wos/woscc/summary/fcd470cc-4585-4263-94c1-152e1f496e85-0dba8195/relevance/1)
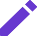


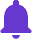

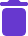


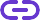
26

Search [Web](https://clarivate.com/legal/copyright/) of Science [8,678,693](https://www.webofscience.com/wos/woscc/summary/82a785c6-02a3-47f6-8ec2-9861f1aaaf18-0dba7e83/relevance/1)

**ALL=(((outcomes) OR (care procedures) OR** Core Collec[tion](https://www.twitter.com/ClarivateAG)

**(organizational structure) OR (resource management) OR** Show editions


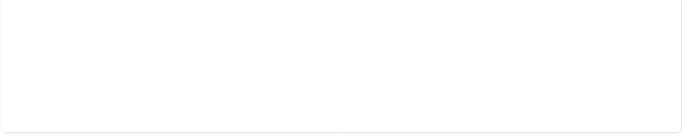

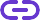

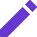

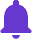

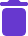

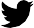

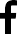


[Training Portal](https://clarivate.com/webofsciencegroup/support/wos/)

[Product Support](https://support.clarivate.com/ScientificandAcademicResearch/s/?language=en_US)

[Privacy Statement](https://clarivate.com/legal/privacy-statement/)

[Newsletter](http://discover.clarivate.com/WebofScienceNewsletter)

**(continuity) OR (eﬀiciency) OR (eﬀicacy) OR (eﬀectiveness) OR (safety) OR (security) OR (accessibility) OR (equity) OR (adaptability) OR (satisfaction) OR (resource mobilization)))**

[Copyright Notice](https://clarivate.com/legal/copyright/) [Cookie Policy](https://clarivate.com/legal/cookie-policy/)

[Terms of Use](http://wokinfo.com/terms)

1:41 PM


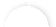

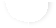


26
